# Stearoyl-CoA desaturase inhibition is toxic to acute myeloid leukemia displaying high levels of the de novo fatty acid biosynthesis and desaturation

**DOI:** 10.1038/s41375-024-02390-9

**Published:** 2024-08-26

**Authors:** Vilma Dembitz, Hannah Lawson, Richard Burt, Sirisha Natani, Céline Philippe, Sophie C. James, Samantha Atkinson, Jozef Durko, Lydia M. Wang, Joana Campos, Aoife M. S. Magee, Keith Woodley, Michael J. Austin, Ana Rio-Machin, Pedro Casado, Findlay Bewicke-Copley, Giovanny Rodriguez Blanco, Diego Pereira-Martins, Lieve Oudejans, Emeline Boet, Alex von Kriegsheim, Juerg Schwaller, Andrew J. Finch, Bela Patel, Jean-Emmanuel Sarry, Jerome Tamburini, Jan Jacob Schuringa, Lori Hazlehurst, John A. Copland III, Mariia Yuneva, Barrie Peck, Pedro Cutillas, Jude Fitzgibbon, Kevin Rouault-Pierre, Kamil Kranc, Paolo Gallipoli

**Affiliations:** 1 Centre for Haemato-Oncology, Barts Cancer Institute, Queen Mary University of London, London, UK; 2 Department of Physiology and Croatian Institute for Brain Research, University of Zagreb School of Medicine, Zagreb, Croatia; 3 The Institute of Cancer Research, London, UK; 4 Division of Cell and Molecular Biology, Imperial College London, London, UK; 5 Francis Crick Institute, London, UK; 6 INSERM U1242, University of Rennes, Rennes, France; 7 Experimental Hematology Lab, IIS-Fundación Jimenez Díaz, UAM, Madrid, Spain; 8 Centre for Cancer Genomics & Computational Biology, Barts Cancer Institute, Queen Mary University of London, London, UK; 9 The University of Edinburgh MRC Institute of Genetics and Cancer, University of Edinburgh, Edinburgh, UK; 10 Department of Experimental Hematology, University Medical Center Groningen, University of Groningen, Groningen, The Netherlands; 11 Centre de Recherches en Cancérologie de Toulouse, Université de Toulouse, Inserm U1037, CNRS U5077, LabEx Toucan, Toulouse, France; 12 Équipe labellisée Ligue Nationale Contre le Cancer 2023, Toulouse, France; 13 University Children’s Hospital and Department of Biomedicine (DBM), University of Basel, Basel, Switzerland; 14 Centre for Tumour Biology, Barts Cancer Institute, Queen Mary University of London, London, UK; 15 Translational Research Centre in Onco-hematology, Faculty of Medicine, University of Geneva and Swiss Cancer Center Leman, Geneva, Switzerland; 16 Modulation Therapeutics, Morgantown, WV USA; 17 Department of Cancer Biology, Mayo Clinic, Jacksonville, FL USA

**Keywords:** Acute myeloid leukaemia, Cancer metabolism, Drug development

## Abstract

Identification of specific and therapeutically actionable vulnerabilities, ideally present across multiple mutational backgrounds, is needed to improve acute myeloid leukemia (AML) patients’ outcomes. We identify stearoyl-CoA desaturase (SCD), the key enzyme in fatty acid (FA) desaturation, as prognostic of patients' outcomes and, using the clinical-grade inhibitor SSI-4, show that SCD inhibition (SCDi) is a therapeutic vulnerability across multiple AML models in vitro and in vivo. Multiomic analysis demonstrates that SCDi causes lipotoxicity, which induces AML cell death *via* pleiotropic effects. Sensitivity to SCDi correlates with AML dependency on FA desaturation regardless of mutational profile and is modulated by FA biosynthesis activity. Finally, we show that lipotoxicity increases chemotherapy-induced DNA damage and standard chemotherapy further sensitizes AML cells to SCDi. Our work supports developing FA desaturase inhibitors in AML while stressing the importance of identifying predictive biomarkers of response and biologically validated combination therapies to realize their full therapeutic potential.

## Introduction

AML is a highly aggressive malignancy of hematopoietic origin. Despite the approval of several novel therapies in the past decade, AML prognosis remains poor with long-term survival rates of about 30%. Development of novel therapeutic approaches for AML is particularly challenging due to high genetic and cellular heterogeneity [1]. Therefore, the identification of specific AML biological features beyond genetic mutations is needed for the development of targeted therapies to improve patient outcomes. Rewired metabolism is one such feature, however, discerning specific metabolic dependencies of malignant cells is crucial to avoid generalized toxicity that often compromises the clinical use of metabolic inhibitors [2].

Fatty acid (FA) metabolism has emerged as a cancer-specific vulnerability in multiple solid cancers [3–5], but its role in hematological malignancies, and specifically AML, is less characterized. In AML most preclinical evidence has focused on the role of fatty acid oxidation (FAO) [6, 7]. However targeting FAO is associated with the risk of cardiac toxicity [8] and the best-characterized FAO inhibitor, etomoxir, proved to be systemically toxic, halting its clinical development [9]. Comparatively, targeting fatty acid synthesis (FAS), particularly stearoyl-CoA desaturase 1 (SCD1, hereafter SCD), the enzyme converting saturated fatty acids (SFA) palmitate and stearate into monounsaturated fatty acids (MUFA) palmitoleate and oleate [10], appears to be more tolerable based on preclinical studies [11]. However, although the role of SCD has been investigated in solid cancer models, translational progress of SCD as a therapeutic target has been hampered by the lack of clinical-grade inhibitors with only one recent report, using the SCD inhibitor YTX-7739 in glioblastoma presenting survival benefit of pharmacological SCD inhibition in an animal solid tumor model [4]. Moreover, in contrast to solid tumors, the role of SCD in hematological malignancies is less clear with few and contradictory reports [12, 13]. In chronic myeloid leukemia (CML), SCD is thought to be a tumor suppressor and its deletion causes acceleration of CML development. Conversely, genetic depletion or pharmacological inhibition of SCD decreases acute lymphoblastic leukemia (ALL) burden in the central nervous system, but has no effect or increases leukemia burden in the bone marrow while the effects on survival were not studied [13]. Specifically in AML, SCD has been shown to play a role in the resistance of AML stem cells to NAMPT inhibitors [14], while SCD inhibition (SCDi) leads to greater sensitivity to FLT3 inhibitors [15]. However, in neither of these reports, the in vivo activity of SCDi was tested to support the translational potential of targeting SCD. Therefore a broader understanding of the significance of SCD levels in AML prognosis and response to therapy, its functional role, and potential as a therapeutic target, including the identification of biological determinants of sensitivity to its inhibition, is required.

Here we address these questions utilizing SSI-4, a clinical-grade SCD inhibitor with a favorable general toxicity profile [16]. We show that SCD expression is prognostic in AML, and its inhibition compromises the viability of AML cell lines and primary samples in vitro and in vivo. Sensitivity to SCD inhibition correlates with higher rates of MUFA synthesis and can be modulated by FA biosynthesis activity. We finally show that SCDi synergizes with standard AML chemotherapy by enhancing DNA damage and their combination further sensitizes AML cells to this novel therapeutic approach.

## Materials and methods

### Detailed methods are included in Supplementary Methods and all reagents used are listed in Supplementary Data 9

#### Cell culture: cell lines and primary human AML patient-derived samples

K562 (ATCC, CCL-243), MOLM-13, MV-4-11, THP-1, HL-60, Kasumi-1, OCI-AML3, TF-1 (Sanger Institute), 293T-Pheonix cells (kind gift of B. Huntly, University of Cambridge) and MS-5 (DSMZ, ACC 441) cells were cultured following ATCC and DSMZ recommendations.

Frozen AML samples from Barts Cancer Institute (*n* = 36) and University Medical Center Groningen (*n* = 25) were retrieved from the respective institute’s biobank thawed and plated in co-culture with MS-5 stromal cells. After treatment with SSI-4, viability was determined using anti-Annexin-V antibody in combination with propidium iodide or DAPI stain. All human samples were obtained and studied after informed consent and protocol approval by Barts Cancer Institute and University Medical Center Groningen Ethical Committees and BCI Tissue Biobank’s scientific sub-committee in accordance with the Declaration of Helsinki.

#### In vivo experiments

The mice strains used in the study were NBSGW and Vav-iCre and were purchased from Jackson Laboratory. *iMLL-AF9* mice were a kind gift of Jürg Schwaller.

Animals were treated orally with 10 or 30 mg/kg SSI-4 in 10% Captisol solution or vehicle control. For experiments involving conventional chemotherapy protocol, it was delivered in a 5-day protocol in which on days 1, 3, and 5 animals intravenously received 1.0 mg/kg doxorubicin and 50 mg/kg cytarabine in the same syringe, and on days 2 and 4 animals intravenously received 50 mg/kg cytarabine. All experiments on animals were performed under UK Home Office authorization.

#### RNA sequencing and analysis

RNA Sequencing and bioinformatics analysis was provided by Novogene UK Company Limited (Cambridge, UK).

#### Glucose labeling

Cells were grown for 24 h in RPMI medium with no glucose, supplemented with 10% FBS, 50 IU/ml penicillin and 50 μg/ml streptomycin, and 2 g/L U-¹³C_6_-Glucose. In analysis, fatty acids containing isotope ¹³C peaks *m* + 0 and *m* + 1 were marked as unlabeled, and the ones containing *m* + 2 and higher as labeled.

#### Metabolomics experiments

For lipidomics analysis, lipid species were extracted using monophasic isopropanol extraction and analyzed using liquid chromatography-mass spectrometry. Lipid annotation was performed with LipiDex software and additional analysis of the lipidomics dataset was performed with the LipidSuite webtool (https://suite.lipidr.org).

For fatty acid profiling, apolar metabolites were isolated from cells using chloroform:methanol extraction, and fatty acids partitioned from polar metabolites by resuspension of dried extracts in chloroform:methanol:water. Data acquisition was performed using gas chromatography-mass spectrometry. Fatty acids were identified and quantified by comparison to authentic standards and ^13^C_1_-lauric acid as an internal standard.

## Results

### SCD levels are prognostic in AML and synthesis of unsaturated fatty acids is more active at points of disease progression and relapse

**Fig. 1:**
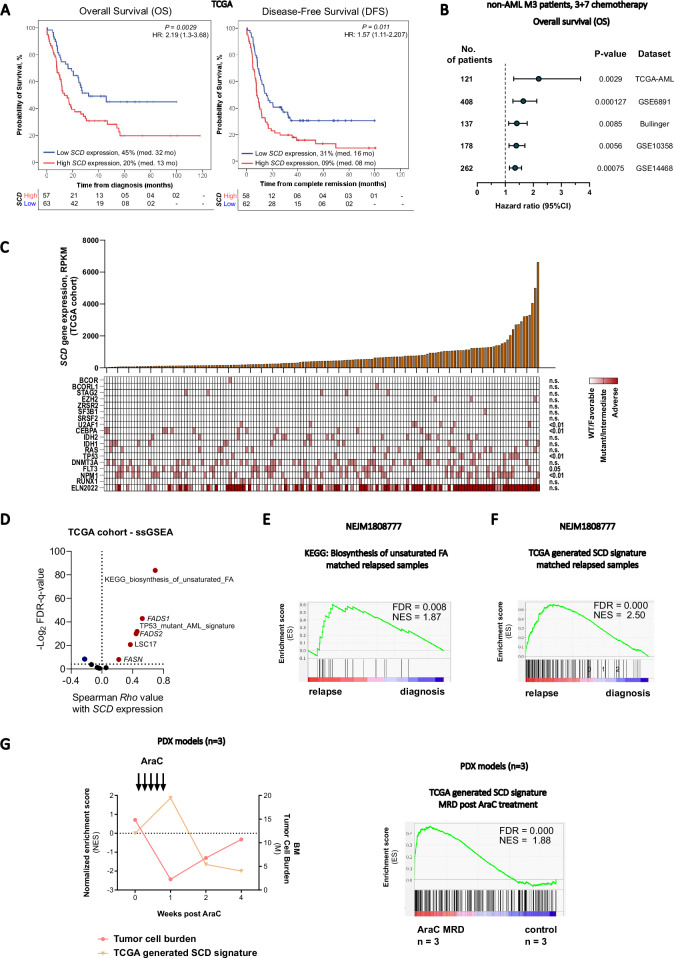
High SCD gene expression levels are prognostic in AML and associated with relapse. **A** Kaplan–Meier curves comparing overall survival and disease-free survival in TCGA AML patient cohort dichotomized after *SCD* expression. The expression level of *SCD* was considered a continuous variable and the Log rank (Mantel–Cox) test was used to determine significance. **B** Forest plot of overall survival analyses considering continuous *SCD* gene expression on several patients’ datasets. Multivariate analysis corrected for confounding variables like age, gender, and ELN prognostic group in all datasets. **C** Oncoprint matrix correlating SCD expression levels with the presence of common mutations in AML and ELN prognostic groups. Kruskal–Wallis test was used for determining significance. **D** Single sample gene set enrichment analysis (ssGSEA) on TCGA cohort in dependency to *SCD* expression. **E** Gene set enrichment analysis (GSEA) for KEGG pathway Biosynthesis of unsaturated fatty acids and **F** TCGA-generated SCD signature in paired diagnosis-relapse primary AML samples (NEJM1808777 dataset). **G** Tumor burden and SCD signature expression in Ara-C-treated PDX models (GSE97631) and GSEA for SCD signature at MRD stage.

Analysis of multiple independent gene expression profiles of newly diagnosed AML samples shows that higher levels of *SCD* expression correlate with significantly decreased survival. *SCD* expression levels remained prognostic even after correcting for age, gender, and European Leukemia Net (ELN) risk group (Fig. 1A, B). This finding was confirmed when analyzing a local cohort of patients with adverse risk AML (Supplementary Fig. 1A). Although *SCD* expression did not correlate with specific ELN risk group, it was higher in patients with specific adverse risk mutations (*U2AF1*, *TP53*) which might partially account for its prognostic role (Fig. 1C). High *SCD* expression correlates with several genes involved in FAS and desaturation such as fatty acid synthase (*FASN)*, fatty acid desaturase 1 and 2 (*FADS1* and *FADS2)* and adverse prognostic features such as *TP53* mutant signatures and the leukemic stem cell signature – LSC17 [17] (Fig. 1D, Supplementary Fig. 1B). Notably genes involved in cholesterol biosynthesis and lipogenesis are part of a recently reported 4-gene prognostic index capable of refining survival predictions in AML patients. Interestingly expression of genes within this signature also correlates with other genes involved in lipogenesis including *SCD* and with TP53 mutation [18]. Indeed a biosynthesis of unsaturated fatty acids (FA) gene signature is enriched in matched post-chemotherapy relapse versus diagnosis in human AML samples (Fig. 1E, Supplementary Fig. 1C), together with a TCGA-generated SCD signature (Fig. 1F). Interestingly, the SCD signature was inversely correlated with tumor burden in Ara-C-treated PDX models [7] and was significantly upregulated at nadir after chemotherapy (minimal residual disease, MRD) (Fig. 1G). Overall these correlative data suggest that synthesis of unsaturated FA associates with poor prognosis and high-risk mutations while also being more active at points of leukemia resistance or progression raising the possibility that SCD is linked with mechanisms regulating sensitivity to chemotherapy and likely explaining its prognostic relevance.

### Pharmacologic inhibition of SCD induces pronounced toxicity in a subset of AML in vitro

**Fig. 2:**
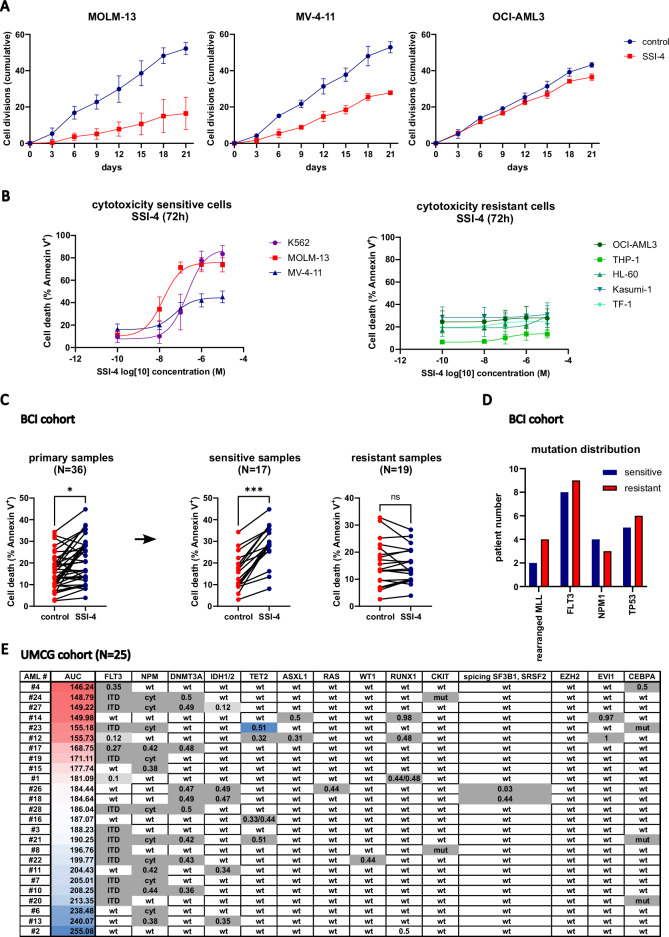
Novel clinical-grade SCD inhibitor SSI-4 induces cell death in a subset of AML samples. **A** Proliferation assay in MOLM-13, MV-4-11, and OCI-AML3 cells treated with SSI-4 (1 µM) for 6 cycles of 72 h in a total duration of 21 days. The number of cell divisions was standardized after the initial plating concentration of 300,000 cells per mL. **B** A panel of eight AML cell lines was treated with SSI-4 (0.01–10 µM) or corresponding vehicle for 72 h. Cells with less than a 10% increase in cell death induction were designated resistant. Results are presented as non-linear regression and data points are mean ± SD. **C** AML primary samples from Barts Cancer Institute (BCI, *n* = 36) were depleted of T-cells and grown in co-culture with irradiated MS-5 cells for 7 days with the addition of SSI-4 (1 µM). Samples with less than 5% increase in cell death induction were designated to the resistant group. **D** Mutation distribution in sensitive and resistant samples across BCI cohort. For a more detailed presentation of the patient’s characteristics please see Supplementary Data 1. **E** A separate AML patients cohort from the University of Groningen Medical Center (UMCG, *n* = 25) was treated with SSI-4 (1 and 10 µM) in co-culture with stroma for 4 days and sensitivity to SSI-4 was expressed as area under the curve (AUC). *p < 0.05, **p < 0.01, ***p < 0.001, ****p < 0.0001.

To test SCD as a therapeutic target, we used the clinical-grade SCD inhibitor SSI-4 against a panel of human AML cell lines. Although SSI-4 treatment decreased viable cell counts in all cell lines tested (Supplementary Fig. 1D), its effects on cell proliferation rate varied (Fig. 2A). Moreover, when cytotoxicity was assessed, SSI-4 induced cell death in several cell lines (K562, MOLM-13, MV-4-11) from now on referred to as sensitive, while others (OCI-AML3, THP-1, HL-60, Kasumi-1, TF-1) were resistant (Fig. 2B).

We validated these data using another SCD inhibitor A939572 (Supplementary Fig. 1E, F). In addition, chronic SCD genetic depletion impairs growth more prominently in SSI-4-sensitive cells (Supplementary. Fig. 1G), but did not induce cell death in standard culture conditions, possibly due to compensatory upregulation of other fatty acid desaturases [19].

To extend the translational relevance of our findings, we tested the cytotoxic effects of SSI-4 on two independent cohorts of primary AML samples in vitro comprising 61 samples in total. Consistent with the findings in AML cell lines, we observed that primary samples grown in stromal co-culture also dichotomized into sensitive and resistant to SCD inhibition, with no clear relation to specific driver mutations (Fig. 2C–E, Supplementary Data 1).

### In vivo SSI-4 treatment does not affect normal hematopoiesis and induces anti-leukemic effects in humanized xenograft models

**Fig. 3:**
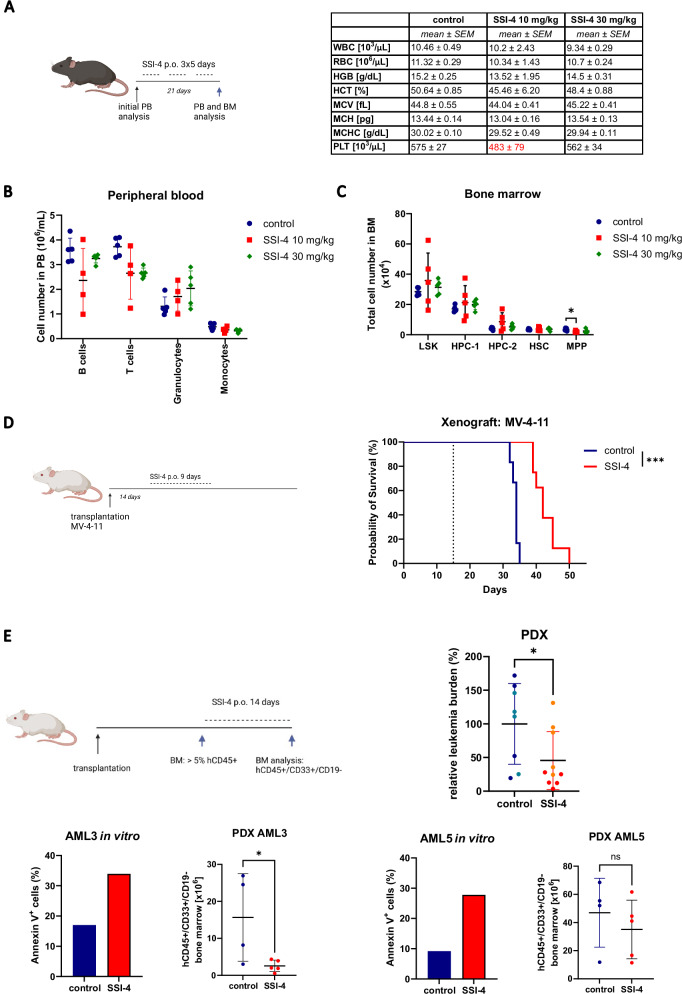
SSI-4 demonstrates no hematopoietic toxicity and displays anti-leukemic effects in vivo in humanized xenografts. **A** C57BL/6 mice (*n* = 15) were treated with 10 and 30 mg/kg SSI-4 or corresponding vehicle orally for a total of 21 days with 2 days break after each 5 days of continuous treatment. PB counts of control or SSI-4 treated mice. WBC – white blood cells, RBC – red blood cells, HGB – hemoglobin concentration, HCT – hematocrit, MCV – mean cell volume, MCH – mean cell hemoglobin, MCHC – mean cell hemoglobin concentration, PLT – platelets. **B** Differential blood counts in peripheral blood of treated animals as determined by flow cytometry. **C** Total numbers of cells in LSK (Lin-Sca-1+c-Kit+), HPC-1 (LSK CD48+CD150−), HPC-2 (LSK CD48+CD150−), HSC (LSK CD48−CD150+), MPP (LSK CD48-CD150−) compartments in the BM isolated from two legs of treated animals. **D** MV-4-11 cells were transplanted into NBSGW mice (*n* = 14). 14 days after transplant animals were treated for 9 days with 10 mg/kg SSI-4 or corresponding vehicle orally. The dotted line represents the start of treatment. Kaplan–Meier curve represents the overall survival of animals treated with SSI-4 and the corresponding vehicle. **E** Two sensitive samples in vitro were transplanted into NBSGW mice. When engraftment of human CD45^+^ cells exceeded 5% in the BM, animals were distributed in groups with equal leukemic burden and treated with 10 mg/kg of SSI-4 or corresponding vehicle orally for 14 days. The total number of human leukemic cells (hCD45^+^hCD33^+^hCD19^−^) isolated from two legs at the end of the experiment was standardized relative to the mean engraftment of individual patient samples at the end of the experiment. Data are mean ± SD. PDX derived from AML3 are presented in darker shades and those derived from AML5 in lighter shades on the dot plot. SSI-4 mediated increase in cell death in vitro and absolute decrease of human leukemic cells in the bone marrow of treated mice for each patient sample is presented in lower panels. *p < 0.05, **p < 0.01, ***p < 0.001, ****p < 0.0001. Mouse illustrations created with BioRender.com.

A favorable general toxicity profile of SSI-4 has been demonstrated in previous animal studies [3, 16], which was confirmed by minor weight loss in our model and only mild side effects consisting of transient hair loss and squinting (Supplementary Fig. 2A, B). However, we also ascertained that SSI-4 had no significant hematopoietic toxicity, as shown by its negligible effects on the peripheral blood (PB) counts and hematopoietic progenitor compartments of treated animals (Fig. 3A–C, Supplementary Fig. 2C).

We then further validated our findings in two murine leukemic cells expressing either *iMLL-AF9* or *Hoxa9/Meis1*. Both antiproliferative and cytotoxic effects were observed in both cell lines following SCDi (Supplementary Fig. 2D–F). Interestingly, while both cell lines demonstrated in vitro sensitivity to SSI-4-mediated cytotoxicity comparable to that of human MV-4-11 cells, in vivo treatment of *iMLL-AF9* model with SSI-4 resulted in induction of differentiation without significant decrease in BM leukemic burden (Supplementary Fig. 2G).

Conversely, despite being only moderately sensitive to SSI-4 mediated cytotoxicity in vitro, 9-day treatment with SSI-4 two weeks after disease initiation significantly prolonged survival in the MV-4-11 cell line derived xenograft (CDX) model (Fig. 3D). We then tested SSI-4 activity in two patient-derived xenografts (PDX) derived from two primary AML samples sensitive to SSI-4 in vitro. Overall a significant decrease in bone marrow (BM) leukemia burden following SSI-4 treatment was observed in both PDX models (Fig. 3E), which was more pronounced in one of the two samples tested, probably due to sample size.

Together these in vivo data show that treatment with SSI-4 is not toxic to the hematopoietic compartment but sensitivity to SCD inhibition is variable, consistent with our observation in vitro. Still, in humanized models, SSI-4 treatment results in decreased leukemia burden and survival prolongation.

### SCD activity dictates sensitivity to SCD inhibition by preventing SFA accumulation and lipotoxicity

**Fig. 4:**
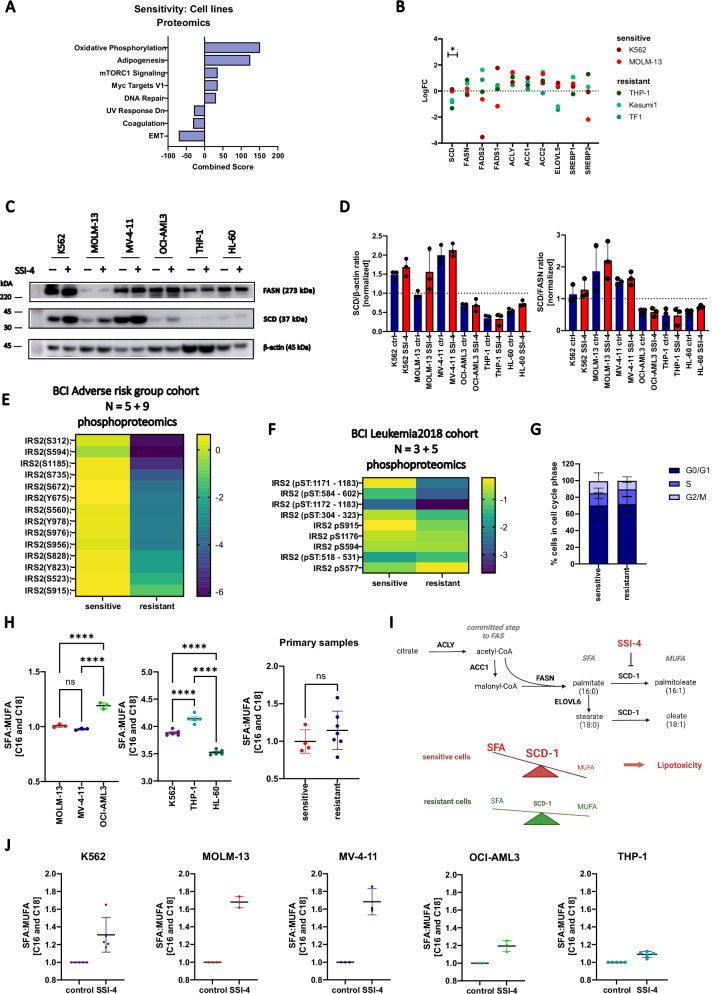
SSI-4 sensitive cells have a greater dependency on de novo MUFA production. **A** Significantly enriched MSigDB signatures in sensitive vs resistant cell lines from the Cancer Cell Line Encyclopedia proteomics dataset ranked by a combined score from *Enrichr* enrichment analysis. Significantly upregulated signatures in sensitive cells are presented on the right-hand side and downregulated signatures on the left-hand side of the graph. **B** Normalized expression of fatty acid synthesis-related proteins in AML cell lines tested. **C** Representative western blot (*n* = 3) of sensitive (K562, MOLM-13, MV-4-11) and resistant (OCI-AML3, THP-1, HL-60) cell lines treated with SSI-4 (1 µM) or vehicle control for 24 h. **D** Densitometric analysis shows SCD expression normalized to ß-actin as a loading control and FASN. **E** Phosphoproteomic analysis of 5 sensitive and 9 resistant AML patients from BCI Adverse prognosis cohort. **F** Independent phosphoproteomic analysis from BCI Leukemia 2018 cohort of 3 sensitive and 5 resistant AML patients. Heatmaps represent log_2_ fold change of phosphorylated sites on IRS2 in sensitive and resistant samples normalized on-target relative intensity. **G** Cell cycle analysis of sensitive (*n* = 7) and resistant (*n* = 8) primary AML samples. **H** SFA/MUFA ratios in sensitive and resistant AML cell lines and primary samples (*n* = 11). The graphs represent the ratio of C16 and C18 saturated (SFA) and monounsaturated fatty acids (MUFA) in independent runs. **I** Schematic representation of de novo fatty acid synthesis pathway and SFA/MUFA imbalance upon SCD inhibition in sensitive and resistant cells. ACLY - ATP-citrate lyase, ACC1 – acetyl-CoA carboxylase, FASN – fatty acid synthase, ELOVL6 - ELOVL fatty acid elongase 6, SCD1 – stearoyl-Co desaturase. **J** SFA/MUFA ratios normalized to control conditions in sensitive and resistant AML cell lines treated with SSI-4 (1 µM) or vehicle control for 24 h.

Since demographic, clinical, or genetic features did not correlate with sensitivity to SCD inhibition in both primary samples and cell lines used (Supplementary Data 1 and 2, Supplementary Fig. 3A), we analyzed publicly available proteomic data [20] for SSI-4 sensitive and resistant cell lines. These showed an enrichment in adipogenesis signature and higher SCD protein expression in sensitive cells (Fig. 4A, B). Western blot analysis confirmed these findings with an increased SCD to fatty acid synthase (FASN) ratio in sensitive cells. This suggests that sensitive cells are less able to tolerate SFA accumulation and display greater dependency on fatty acid desaturation regardless of basal levels of FAS activation (Fig. 4C, D, Supplementary Fig. 3B). Additionally, sensitivity to SCD inhibition did not correlate with uptake of external lipids, or expression of lipid transporters CD36 and LDLR (Supplementary Fig. 3C), both previously identified as independent prognostic factors in AML [21, 22], highlighting the importance of de novo fatty acid synthesis.

To further understand features associated with SSI-4 sensitivity we used (phospho-) proteomic and transcriptomic analysis in a proportion of our primary AML samples cohort. In primary samples, SCD expression was undetectable by proteomic analysis however a correlative trend between SCD mRNA expression and cytotoxicity in response to SSI-4 was observed (Supplementary Fig. 3D). In addition, while we did not identify a specific biomarker using transcriptomic or proteomic approaches (data not shown), phosphoproteomic data from two separate analysis on different primary AML samples subsets showed significantly higher levels of phosphorylated-insulin receptor substrate 2 (IRS2) in sensitive cells (Fig. 4E, F). IRS2 is a downstream target of receptor tyrosine kinases (RTK) and has been shown to specifically regulate the insulin-like growth factor-1 (IGF-1) autocrine production and signaling in AML [23]. The activation of RTK was further supported by increased phosphorylation of AKT2 and PLEKHG3 in sensitive cells (Supplementary Fig. 3E). Interestingly, sensitive cell lines were insulin resistant (Supplementary Fig. 3F), consistent with greater constitutive activation of pathways downstream of RTK. Interestingly previous studies have shown that lower response to insulin is associated with increased susceptibility to metabolic inhibitors [24]. Additionally, in sensitive cell lines, SSI-4 decreases phosphorylation of IRS2 downstream targets, Akt and p70S6K, indicative of a functional role of this signaling pathway in SSI-4-mediated effects although more work is needed to elucidate this further (Supplementary Fig. 3G). Interestingly, increased activation of signaling downstream of RTK in primary AML did not correlate with faster progression through cell cycle (Fig. 4G) suggesting that sensitivity is not linked to a more proliferative phenotype, but potentially to the known role of RTKs in regulation of de novo fatty acid synthesis and desaturation [25].

Indeed, fatty acid quantitation showed that the SFA/MUFA ratio is significantly higher in resistant cell lines when compared to sensitive ones, with the HL-60 cell line acting as an outlier probably due to its phenotypic similarities to acute promyelocytic leukemia (Fig. 4H). Similar trend in SFA/MUFA ratio between sensitive and resistant cells could also be observed in primary AML samples, as SFA/MUFA ratio was inversely correlated to cell death induction (Fig. 4H, Supplementary Fig. 3H). Higher dependency on SCD activity in sensitive cells is further confirmed with the expected relative increase in ratio upon SSI-4 treatment being lower in the resistant OCI-AML3 and THP-1 compared to the sensitive cells (Fig. 4J).

SCD is an oxygen-dependent enzyme [26] and interestingly hypoxic conditions phenocopy the effects of SCDi both on the levels of SFA and MUFA and viability in sensitive and resistant cells (Supplementary Fig. 4A, B). Such inhibitory effect results in a compensatory increase in SCD expression in hypoxic conditions making sensitive cells in particular even more susceptible to SCDi while having no effect on resistant cells (Supplementary Fig. 4C, D). This was confirmed in SCD genetically depleted cells as hypoxic conditions induced cell death specifically in sensitive cells previously shown to be resistant to SCD depletion-induced citotoxicity in normoxic conditions (Supplementary Fig. 4E, F, Supplementary Fig. 1G). Together these data suggest that SSI-4 sensitive cells have a more active de novo fatty acid desaturation. Upon SCD inhibition FAS becomes uncoupled from desaturation thus causing an imbalance between SFA and MUFA levels to a degree able to trigger lipotoxicity [27] and cell death (Fig. 4I).

### Sensitivity to SCD inhibition in AML cells can be modulated by regulating FAS activity

**Fig. 5:**
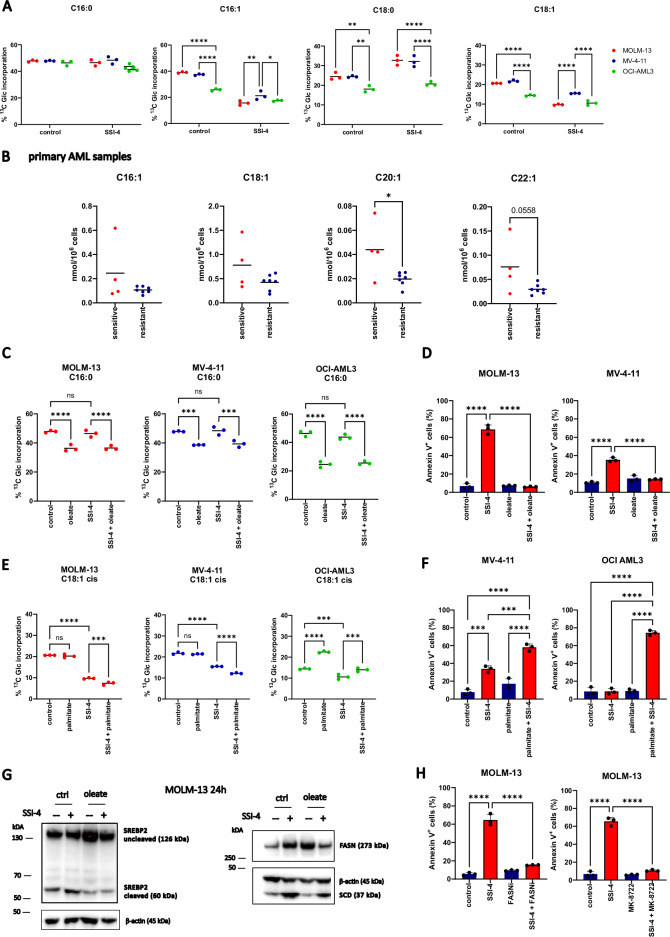
MUFA production and levels regulate sensitivity to SSI-4 by modulating the de novo fatty acid synthesis pathway. **A** MOLM-13, MV-4-11, and OCI-AML3 were grown in medium supplemented with U-^13^C_6_-Glucose (2 g/L) and treated with SSI-4 (1 µM) or vehicle control for 24 h. Graphs represent the percentage of ^13^C-glucose incorporation in palmitate (C16:0), stearate (C18:0), palmitoleate (C16:1), and oleate (C18:1). **B** MUFA levels in sensitive (*n* = 4) vs resistant (*n* = 7) primary AML samples. **C**, **E** MOLM-13, MV-4-11, and OCI-AML3 were labeled with U-^13^C_6_-Glucose (2 g/L) and treated for 24 h with SSI-4 (1 µM) or vehicle control with or without the addition of oleate (100 µM) or palmitate (100 µM). Graphs represent the percentage of ^13^C-glucose incorporation in palmitate (C16:0) and oleate (C18:1) (**D**, **F**) MOLM-13, MV-4-11, and OCI-AML cells were treated for 72 h with SSI-4 (1 µM) with or without the addition of oleate (100 µM) or palmitate (100 µM). **G** Representative western blots (*n* = 3) of MOLM-13 cells treated for 24 h with SSI-4 (1 µM) or vehicle control with or without the addition of oleate (100 µM). **H** MOLM-13 cells were treated for 72 h with SSI-4 (1 µM) with or without the addition of FASN inhibitor Fasnall (20 µM) or MK-8722 (10 µM). Cell death induction was determined by Annexin-V expression. Data are mean ± SD. *p < 0.05, **p < 0.01, ***p < 0.001, ****p < 0.0001.

In order to validate the increased dependency of SSI-4 sensitive cells on fatty acid synthesis/desaturation and the postulated mechanism of lipotoxicity induction in response to SCD inhibition, we performed gas chromatography-mass spectrometry (GC/MS) experiments tracing uniformly labeled ^13^Carbon (U-^13^C_6_) glucose incorporation into FA. In sensitive cells, MUFA biosynthesis (16:1, C18:1) was higher and more responsive to SCD inhibition (Fig. 5A, Supplementary Fig. 5A, B). Supporting and extending these findings we observed that sensitive AML primary samples display higher levels of MUFA in comparison to resistant ones (Fig. 5B), confirming greater dependency on FA desaturation in these cells.

We also noticed increased levels of labeled SFA stearate (C18:0) in sensitive cells upon SSI-4 treatment (Fig. 5A), which could be explained by both decreased desaturation and conversion to oleate and/or increased production. To then test whether SCD activity and oleate levels regulate de novo fatty acid synthesis pathway in AML, we grew cells in the presence of oleate and noted decreased production of both SFA and MUFA (Fig. 5C and Supplementary Fig. 5A, B) which also correlated with a complete rescue of SSI-4-mediated decrease in viability, supporting the on-target efficacy of SSI-4 (Fig. 5D). Conversely, addition of palmitate in non-toxic concentration increased FAS and oleate production mostly in resistant cells (Fig. 5E and Supplementary Fig. 5A, B). This suggests that baseline MUFA production in resistant cells is below its potential maximum and can be upregulated following palmitate supplementation. Although exogenous palmitate can still be detoxified by desaturation both in sensitive and resistant cells, as shown by total levels of labeled and unlabeled MUFA (Supplementary Fig. 5B), in palmitate-rich conditions, SCD inhibition results in a large increase in the SFA/MUFA ratio. This is true even in resistant cells that do not display such strong imbalance upon treatment with SSI-4 alone (Fig. 4H, Supplementary Fig. 5C) and causes increased sensitivity to SSI-4 in both sensitive and resistant cells in the presence of palmitate (Fig. 5F). It is worth noting here that induction of lipotoxicity in AML cells is likely a dynamic process not related with exceeding a single specific SFA/MUFA ratio level. The SFA/MUFA threshold inducing lipotoxicity is likely to vary in different cell lines as a consequence of their ability to tolerate specific levels of SFA/MUFA in different settings. This in turn dictates their dependency on SCD activity. Indeed palmitate accumulation reduces live cell numbers already after 24 h when combined with SCDi, while SCDi alone requires longer exposure and cumulative increase in SFA/MUFA ratio to induce cytotoxicity in cells dependent on SCD. Moreover, if desaturase activity is sustained, exogenous palmitate does not result in cell death, even though it acutely increases the SFA/MUFA ratio (Fig. 5F, Supplementary Fig. 5C, D) [19]. However, the decrease in de novo MUFA production in response to SSI-4 directly correlates with cell toxicity (Supplementary Fig. 5E) confirming again higher reliance of sensitive cells on SCD activity.

Our U-^13^C_6_ labeling experiments demonstrate that changes in MUFA and SFA levels affect sensitivity to SCD inhibition via modulation of total FAS. To clarify the mechanistic underpinning of this observation, we analyzed changes in the regulatory pathways of FAS in sensitive cells. Consistent with the observed increase in the FAS rate, SCD inhibition reduced activation of AMPK (Supplementary Fig. 6A), thereby relieving its inhibitory role on cleavage and consequent activation of SREBP2 (Fig. 5G), a key transcription factor modulating FAS enzymes expression [28, 29]. As expected following SREBP2 activation, we observed increased levels of FASN, SCD, and total acetyl-Coa carboxylase (ACC) which were reversed by the addition of oleate, thus confirming that MUFA levels relieve SCD inhibition toxicity through downregulation of FAS (Fig. 5G, Supplementary Fig. 6B). Conversely, the AMPK activator MK-8722 decreased both SREBP2 cleavage and expression of SCD and FASN (Supplementary Fig. 6C). Although SREBP2 has mostly been described as a regulator of cholesterol synthesis, while FAS is generally under the regulation of SREBP1 [29], in our system we observed more consistent effects on SREBP2 following SCD inhibition and MUFA addition. Conversely, we did not see a change in SREBP1 cleavage in response to SSI-4, with or without the addition of oleate, even though AMPK activation with MK-8722 decreased SREBP1 cleavage as expected (Supplementary Fig. 6D). These data suggest that FAS in AML cell lines is prominently regulated by SREBP2, consistent with other models [30].

Decreasing FAS by inhibition of either FASN or ACC or via AMPK activation abolished SSI-4 mediated toxicity (Fig. 5H, Supplementary Fig. 6E). Interestingly, analysis of the Depmap dataset shows that SCD dependency inversely correlates with the expression levels of both *FASN* and *ACACA* (ACC) across all cancer cells lines and particularly AML ones (Supplementary Fig. 6F). Overall these data confirm that modulation of FAS impacts sensitivity to SCD inhibition.

### Cell death in response to SSI-4 is mediated by lipid oxidative stress, integrated stress response, and activation of apoptotic machinery

**Fig. 6:**
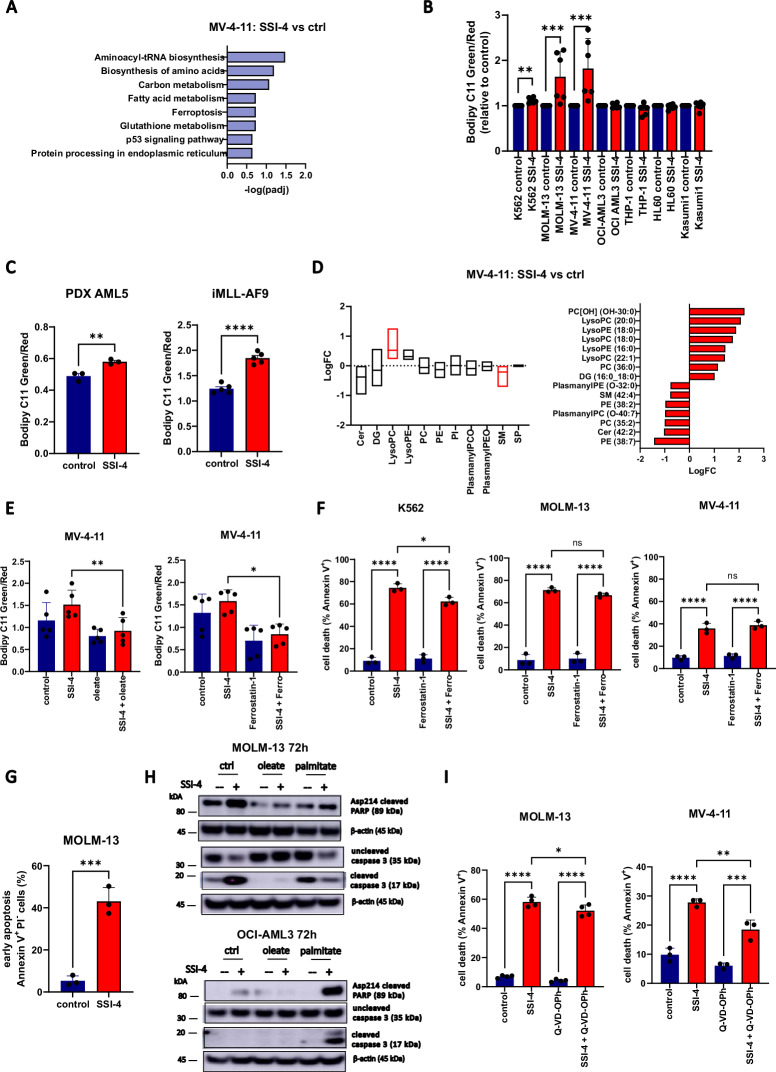
SSI-4 treatment induces both an increase in lipid peroxidation and activation of apoptotic machinery. **A** Significantly enriched KEGG pathway signature in MV-4-11 cells treated with SSI-4 (1 µM) or vehicle control for 24 h. **B** Sensitive K562, MOLM-13, MV-4-11 and resistant OCI-AML3, THP-1, HL-60 and Kasumi-1 cell were treated for 24 h with SSI-4 (1 µM) or vehicle control. Lipid peroxidation was measured using Bodipy C11. **C** Lipid peroxidation determined using Bodipy C11 in the PDX model derived from patient sample AML5 and in the murine AML model *iMLL-AF9* after oral treatment with SSI-4 (10 mg/kg). **D** Lipidomics analysis on MV-4-11 cells treated for 24 h with SSI-4 (1 µM) or vehicle control. The upper graph represents enrichment analysis per lipid groups of treated cells vs. control (Q1–Q3 with line at median value) with significant lipid groups marked in red. The lower graph represents significant differentially expressed individual lipids with upregulated lipids presented on the right-hand side and downregulated lipids on the left-hand side of the graph. Red bars: padj <0.05. **E** Rescue of lipid peroxidation induction in response to SSI-4 (1 µM, 24 h) using oleate (100 µM), as well as lipid peroxidation inhibitors ferrostatin-1 (5 µM) in MOLM-13 cells. **F** K562, MOLM-13, and MV-4-11 cells were treated for 72 h with SSI-4 (1 µM) or vehicle control with or without the addition of Ferrostatin-1 (5 µM). **G** MOLM-13 cells in early apoptosis (Annexin-V^+^/PI^−^) after 72 h treatment with SSI-4 (1 µM). **H** Representative western blots (*n* = 3) of MOLM-13 and OCI-AML3 cells treated for 72 h with SSI-4 (1 µM) or vehicle control with or without the addition of oleate (100 µM) or palmitate (100 µM). **I** MOLM-13 and MV-4-11 cells were treated for 72 h with SSI-4 (1 µM) or vehicle control with or without the addition of Q-VD-OPh (50 µM). Cell death induction was determined by Annexin-V expression. Data are mean ± SD. *p < 0.05, **p < 0.01, ***p < 0.001, ****p < 0.0001.

Transcriptomic analysis of SSI-4 treated cells confirmed the regulatory role of oleate levels on the rate of FAS but also identified oxidative stress-associated pathways (ferroptosis, glutathione metabolism) and integrated stress/endoplasmic reticulum (ER) stress response as potential downstream mechanisms leading to cell death (Fig. 6A and Supplementary Fig. 7A).

Consistent with SCD role in protection against oxidative stress and lipid peroxidation [15], sensitive cells displayed a specific increase in lipid peroxidation as measured by Bodipy C11 staining upon treatment with SSI-4 (Fig. 6B). A similar effect was observed in cells with downregulated SCD in hypoxic condition where lipotoxicity is present (Supplementary Fig. 7B). Furthermore, in the in vivo models where SSI-4 mediated toxicity was less pronounced, we still observed lipid peroxidation induction in leukemia cells derived from treated animals suggesting these cells might be primed for aberrant oxidative stress (Fig. 6C). Lipidomic analysis confirmed that lysophospholipids which have lost their polyunsaturated tail, a known marker of lipid peroxidation [31], are the most enriched lipid class in response to SSI-4 (Fig. 6D). This pattern was completely abrogated by the addition of oleate (Supplementary Fig. 7C). However, despite reducing peroxidation to the same extent of oleate (Fig. 6E, Supplementary Fig. 7D), lipid peroxidation inhibitors only partially rescued or failed to rescue sensitive cells from SSI-4-mediated cell death (Fig. 6F, Supplementary Fig. 7E). Reversal of lipid peroxidation is thus not sufficient to prevent cell death induced by SCD inhibition.

Lipid peroxidation can be a by-product of ER stress [32] and SCD is essential for ER homeostasis [33] given that SFA/MUFA imbalance is known to trigger ER stress [34]. Based on our transcriptomic data (Supplementary Fig. 7A), we interrogated the three arms of the ER response pathway. We noticed a substantial increase in targets downstream of PERK, CHOP and ATF4, a moderate increase in IRE1 and IRE1-associated targets, spliced and total XBP1, and no effects on the expression of ATF6 (Supplementary Fig. 7F). In accordance to that, PERK inhibitor GSK2656157 rescued SSI-4-mediated cytotoxicity, while IRE1 inhibitor 4µ8c demonstrated only milder cytoprotective effects at higher doses and ATF6 inhibitor Ceapin A7 had no effects (Supplementary Fig. 7G). The PERK pathway is a known regulator of apoptosis [35] and SSI-4 treatment induced accumulation of apoptotic marker Annexin-V and activation of apoptotic machinery in both sensitive cells and resistant ones grown in the presence of palmitate (Fig. 6G, H). Still, in contrast to oleate supplementation, co-treatment with pan-caspase inhibitor Q-Vd-OPh again resulted in a significant, but only partial rescue of SSI-4-mediated cell death (Fig. 6I).

Overall these data show that the lipotoxic reaction in response to SSI-4 cannot be reduced to the activation of a single effector death mechanism and that SCD inhibition acts as a pleiotropic trigger which can activate several cell death modes concurrently, thus explaining the detection of both lipid peroxidation and apoptosis markers [36]. Consistent with this, inhibiting any of these cell death mechanisms independently did not completely rescue the cytotoxic effects of SSI-4 supporting their functional redundancy.

### SSI-4 combination with doxorubicin-based chemotherapy is synergistic and prolongs survival in murine AML models with lower sensitivity to single-agent SSI-4

**Fig. 7:**
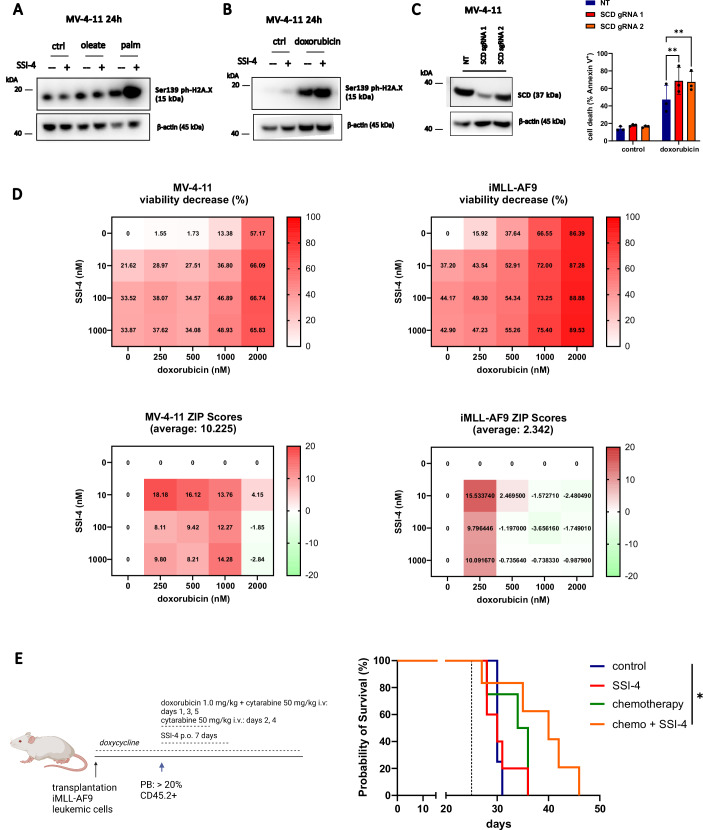
Lipotoxicity increases DNA damage and sensitizes SSI-4 treated cells to DNA-damage-inducing chemotherapy both in vitro and in vivo. **A**, **B** Representative western blots (*n* = 3) of MV-4-11 cells treated with SSI-4 (1 µM) or vehicle control with or without the addition of oleate (100 µM), palmitate (100 µM) or doxorubicin (1 µM) for 24 h. **C** MV-4-11 non-targeting (NT) gRNA, SCD gRNA 1, and SCD gRNA 2 were treated for 72 h with doxorubicin (1 µM). Cell death induction was determined by Annexin-V expression. **D** MV-4-11 and leukemic iMLL-AF9 cells were treated for 72 h with growing concentrations of SSI-4 and doxorubicin. Synergy was determined by the Bliss coefficient (ZIP Score > 10 indicates synergism). Viable cells were determined as Annexin-V^-^/Zombie^-^. **E** CD45.2^+^ leukemic *iMLL-AF9* cells were transplanted into CD45.1^+^ NBSGW mice (*n* = 18). When leukemic burden in PB reached 20%, animals were treated for 7 days with 10 mg/kg SSI-4 or corresponding vehicle orally with or without conventional chemotherapy protocol. The dotted line represents the start of treatment. Kaplan–Meier curve represents the overall survival of animals treated with SSI-4 and corresponding vehicle with our without conventional chemotherapy. Data are mean ± SD. *p < 0.05, **p < 0.01, ***p < 0.001, ****p < 0.0001.

Lipid peroxidation is the most consistent phenotype observed in response to SSI-4 across all AML models tested and is known to induce DNA damage [37]. Besides, the role of SCD inhibition in modulating DNA damage repair via downregulation of RAD51 has already been reported [33]. In MV-4-11 cells, a strong lipotoxic phenotype upon combined treatment with palmitate and SSI-4 induced DNA damage as measured by phosphorylated histone H2A.X (Fig. 7A). This prompted us to assess the therapeutic potential of SSI-4 combination with the DNA-damaging chemotherapeutic doxorubicin. Indeed, SSI-4 increased doxorubicin-induced DNA damage (Fig. 7B) with similar effects on lipid peroxidation (Supplementary Fig. 8A). Moreover SCD depletion resulted in growth disadvantage in the presence of doxorubicin and increased sensitivity to doxorubicin-induced cytotoxicity (Fig. 7C, Supplementary Fig. 8B). However, we did not observe an increase in 4-hydroxynonenal levels in response to either SSI-4 or doxorubicin indicating that the effect of SCDi on DNA damage in this model is not linked to lipid peroxidation and oxidative stress (Supplementary Fig. 8C) but likely linked to its reported effects in modulating the expression levels of protein involved in the DNA damage response [33]. Consistent with this, lipid peroxidation inhibitor did not rescue the combined effects of doxorubicin and SSI-4 (Supplementary Fig. 8D). Conversely, correcting imbalances in the SFA/MUFA ratio and induction of lipotoxicity following SCDi by limiting palmitate production through FASN inhibition or AMPK activation reduces sensitivity to doxorubicin alone or in combination with SSI-4 (Supplementary Fig. 8E).

We detected synergism between SSI-4 and doxorubicin in MV-4-11 cells at the majority of dose combinations, while in *iMLL-AF9* cells, which displayed greater sensitivity to doxorubicin, synergy was evident when doxorubicin was applied in lower concentrations (Fig. 7D). Moreover, analysis of the BeatAML dataset showed that higher *SCD* expression correlates with reduced sensitivity in vitro to cytarabine, an antimetabolite known to cause DNA damage and used in combination with anthracyclines to treat AML patients (Supplementary Fig. 8F). Finally, to validate these findings in vivo we used an aggressive model of fully established AML representative of the scenario routinely encountered in clinic. In contrast to the CDX model where treatment was administered early upon transplantation of leukemic cells (Fig. 3D), animals transplanted with leukemic *iMLL-AF9* cells reached an average leukemic blasts infiltration in PB of 20% before treatment (Supplementary Fig. 8G), a common criterion for AML diagnosis. Similarly to what we observed in the previous experiment using the *iMLL-AF9* model (Supplementary Fig. 2D), 14-day treatment with SSI-4 alone upon aggressive disease establishment was not sufficient for a survival prolongation, and significant survival prolongation could also not be seen in animals treated just with chemotherapy consisting of doxorubicin and cytarabine, a protocol mimicking the standard intensive chemotherapy used in patients. However, consistent with the in vitro findings, combining SSI-4 treatment with chemotherapy, significantly prolonged the survival of treated animals (Fig. 7E). Together these results demonstrate that, in an AML setting with decreased sensitivity to SSI-4 alone, SCD inhibition augments the efficacy of standard AML chemotherapy, likely by enhancing their ability to induce DNA damage. Mouse illustration created with BioRender.com.

## Discussion

In this work, we sought to understand AML metabolic reliance on FAS to uncover novel therapeutic vulnerabilities. Consistent with observations in several solid cancers [38, 39], high SCD expression is an adverse prognostic marker in AML. The prognostic role of SCD is likely due to its association with sensitivity to chemotherapy given that biosynthesis of unsaturated fatty acids is enriched in relapsed and chemo-refractory patients. These observations emphasize the potential of SCD inhibition as a treatment strategy in AML also when combined with standard chemotherapy as supported by our data. The greatest challenge in targeting SCD has been the lack of available clinical-grade inhibitors [11]. SSI-4 is under clinical development for hepatocellular carcinoma [3], and in our study demonstrated potent anti-leukemic effects in vitro and in vivo on roughly half of AML samples and models tested while showing no general or hematopoietic toxicities. Our report is, to the best of our knowledge, the first to demonstrate single-agent activity in vivo and survival prolongation in several AML animal models of a clinical-grade SCD inhibitor.

Although specific mutations can dictate selective metabolic vulnerabilities [40], metabolic dependencies can be present across multiple genetic backgrounds as a phenotype bottleneck i.e. a state essential for continued tumorigenesis. This can be advantageous as metabolic vulnerabilities can be exploited in a larger proportion of patients in a mutation-agnostic manner but also highlights the challenge to identify determinants of sensitivity. This is particularly crucial for metabolic inhibitors as they often target pathways central to the function of normal cells/tissues.

We observed that AML samples clearly dichotomized in sensitive and resistant to SCD inhibition, a pattern also observed in glioblastoma and melanoma [4, 41]. AML sensitivity to SSI-4 was not related to mutational background, instead, sensitive cells mostly displayed both greater de novo MUFA production and higher MUFA levels. Sensitive cells’ dependency on FA desaturation caused a greater SFA/MUFA imbalance upon SCD inhibition resulting in lipotoxicity. Interestingly, SCD appears to be a regulatory nexus of de novo FAS in AML cells, because oleate decreases FAS both in resistant and sensitive cells, rescuing SSI-4-mediated toxicity both by replenishing the MUFA pool, but also preventing SFA production. Similar effects were observed in pancreatic duct adenocarcinoma (PDAC) cells where exposure to oleate also decreased FA production, irrespective of SCD inhibition, and inhibition of SFA production rescued toxicity of SCD inhibition, consistent with our model [42]. It is therefore clear that to drive cytotoxicity via SCD inhibition a significant imbalance between SFA/MUFA needs to be generated. This could be also achieved by modulating the diet, either through a palmitate-rich or a caloric-restricted diet, which creates a dependency on FAS and reduces SCD levels [42] and will be the focus of future work. Conversely, therapeutic interventions that inhibit FAS might reduce the efficacy of SCD inhibition and should be avoided in this setting.

While we did not detect a transcriptional signature of sensitivity, we noted that sensitive primary AML samples displayed increased phosphorylation of IRS2, a direct downstream target of insulin and growth factor receptors [43]. Insulin is a known regulator of SCD expression [44], and sensitivity to SCD inhibition in glioblastoma has been linked to increased ERK phosphorylation [4], also a downstream target of RTK signaling [45]. Although our data indicate that sensitivity to SCD inhibition might correlate with levels of RTK signaling, possibly through its ability to modulate FAS, further work on larger patient cohorts is required to confirm this as a predictive biomarker of response. When assessing sensitivity to SCD inhibition, the limitations of AML modeling should be noted. Namely, in vitro cultures do not represent in vivo metabolic conditions with accuracy because standard serum-complemented media are rich in glucose and scarce in both MUFA and polyunsaturated fatty acids (PUFA) resulting in discrepancies in fatty acid profiles in cultured cells when compared with in vivo conditions [46]. This might impart a more prominent dependency on SCD as cells rely more on de novo FA desaturation activity thus enhancing its role as a marker of sensitivity in the culture system. Conversely, SCD expression in primary samples showed a less stringent correlation to sensitivity to SCD inhibition. Overall based on our data in both primary samples and cell lines, we conclude that FA desaturation activity mostly explains the biological basis for the induction of toxicity in response to SCD inhibition rather than acting as a robust biomarker of response.

The exact mechanism through which lipotoxicity induces cell death remains ill-defined. A previous report ascribed palmitate-induced toxicity to the induction of ER stress [34]. Conversely, reduction of MUFAs is a known inducer of ferroptotic cell death [47]. In response to SCD inhibition, we observed pleiotropic effects causing both increased ER stress with activation of transcription factor DDIT3/CHOP and apoptotic machinery and elevated lipid peroxidation which is a hallmark of ferroptosis [31]. However, inhibition of each of these pathways alone could achieve only a partial rescue of SSI-4-mediated cell death, indicating their functional redundancy. Indeed it has already been shown in glioma cells that SCD inhibition results in distinct downstream effects [27] and both apoptotic and ferroptotic cell death pathways are triggered in response to SCD inhibition in ovarian cancer [36]. These conclusions are further supported by the observation that oleate supplementation, which can fully rescue the viability of SSI-4 treated cells, acts in parallel on FAS, lipid peroxidation, ER stress, and apoptosis, in accordance with its already known ability to rescue both apoptotic and ferroptotic cell death in response to SFA accumulation [48]. Moreover, consistently increased peroxidation markers in response to SCD inhibition followed by the lack of rescue in response to ferroptosis inhibitors, together with complete abrogation of SSI-4-mediated toxicity by FASN inhibition, points to the potential role of ER-associated ROS production in SSI-4-treated cells [49] and suggests that increased peroxidation is mostly a by-product of ER stress rather than a marker of significant ferroptosis induction in our system.

Interestingly induction of lipid peroxidation following SCD inhibition was maintained in vivo even in models less sensitive to single-agent SSI-4 where only a milder decrease in leukemic burden was observed. As lipid peroxidation can induce DNA damage, this prompted us to postulate that, even in cases less sensitive to SCD inhibition, SSI-4 treated leukemic cells are primed for a second cytotoxic hit with DNA-damage-inducing chemotherapy [50]. Indeed, in our models and consistent with what was observed also in glioblastoma [4], lipotoxicity increased DNA damage, although, surprisingly, the effect was not mediated through induction of oxidative stress but likely secondary to the previously described effects of SCD inhibition on DNA damage response machinery [33]. As predicted, we then observed synergy between SCD inhibition and doxorubicin in vitro, and the combination of SSI-4 with conventional AML chemotherapy in vivo significantly prolonged survival in the AML model that demonstrated decreased sensitivity to SCD inhibition alone. These findings highlight that lethal metabolic bottlenecks can be unmasked or enhanced by the action of already approved therapeutic interventions and metabolic vulnerabilities can be fully exploited via synergistic combination therapies [15, 51, 52].

In conclusion, our findings support the efforts of devising new treatment approaches in AML focusing on the metabolic axis of MUFA synthesis. Going forward, as will be the case for most metabolic inhibitors, further research on the identification of predictive biomarkers of response and novel combination approaches, with either other therapies or dietary interventions, is essential for enhancing the efficacy and fully realizing the potential of targeting this axis in AML.

## Supplementary information

Supplemental document

Supplementary Data 1. Patient samples characteristics

Supplementary Data 2. Cell lines characteristics

Supplementary Data 3. Lipidomics analysis

Supplementary Data 4. Fatty acid profiling cell lines

Supplementary Data 5. Fatty acid profiling primary samples

Supplementary Data 6. SCDi vs control DEG

Supplementary Data 7. SCDi oleate vs SCDi DEG

Supplementary Data 8. Enriched datasets

## Data Availability

All reagents and materials in this study are listed in Supplementary Data 9. All cell lines generated in this study can be obtained upon request. The RNA-sequencing data generated in this study have been deposited in the ArrayExpress database and are available at E-MTAB-13174. Data from lipidomics analysis and free fatty acid profiling are available in Supplemental Data 3, 4, and 5. DNA sequencing, RNA sequencing, proteome, and phosphoproteome data on BCI primary AML samples were derived from previously published studies from Barts Cancer Institute [53, 54]. Publicly available clinical and transcriptomic data of five adult AML cohorts whose patients were treated with intensive chemotherapy were used to investigate the prognostic role of SCD expression: AML TCGA (data obtained from https://www.cbioportal.org/), GSE6891, GSE425 (Bullinger), GSE10358, GSE14468. Normalized gene expression data were retrieved from the Gene Expression Omnibus (GEO) database (www.ncbi.nlm.nih.gov/geo/). SCD signature was generated by dichotomizing patients in the TCGA dataset in high and low-expressing samples based on median expression. Dataset GSE97631 was used to determine SCD signature expression in the MRD stage. Data 10.1056/NEJMoa1808777 (NEJM1808777) [55] and GSE66525 were used to perform comparative RNASeq analyses on paired diagnosis-relapse samples in human cohorts and murine models. SCD signature was generated by dichotomizing patients in the TCGA dataset in high and low-expressing samples based on median expression. Dataset GSE97631 was used to determine SCD signature expression in the MRD stage. BeatAML dataset (data obtained http://www.vizome.org/), was used to determine the association of SCD expression with sensitivity to cytarabine ex vivo. Data from manuscript 10.1016/j.cell.2019.12.023 were used for proteomics comparison of sensitive and resistant cell lines. The normalization method used is described in the manuscript [20]. Gene set enrichment analysis was performed using Molecular Signature Database (MSigDB) gene sets. All code and data analyses are available upon request.
