# Supplementary material for: Stearoyl-CoA desaturase inhibition is toxic to acute myeloid leukemia displaying high levels of the de novo fatty acid biosynthesis and desaturation
Source: Leukemia. 2024 Aug 26;38(11):2395–409. doi: 10.1038/s41375-024-02390-9 (PMC11518998; doi:10.1038/s41375-024-02390-9)
Supplement: Supplementary file 1 — Supplemental document [file 41375_2024_2390_MOESM1_ESM.docx]

**SUPPLEMENTAL DOCUMENT**

**SUPPLEMENTAL METHODS**

*Cell culture*

K-562 (ATCC, CCL-243), MOLM-13, MV-4-11, THP-1, HL-60, Kasumi-1, OCI-AML3, TF-1 (Sanger Institute), 293T-Phoenix cells (kind gift of B. Huntly, University of Cambridge) and MS-5 (DSMZ, ACC 441) cells were cultured following ATCC and DSMZ recommendations. Cell lines used were STR typed and regularly checked for Mycoplasma contamination.

For the purpose of exogenous addition of fatty acids, sodium oleate (Sigma-Aldrich) was dissolved in sterile water and conjugated with fatty acids-free bovine serum albumin (Sigma-Aldrich) in ratio 1:2 at 37 °C for 20 minutes. Sodium palmitate (Sigma-Aldrich) was dissolved in 100% ethanol at 75 °C for 10 minutes and conjugated with fatty acids-free bovine serum albumin in ratio 1:3 at 37 °C for 20 minutes. Dissolved sodium oleate was stored at -20°C, and dissolved sodium palmitate was prepared fresh.

*Primary human AML patient derived samples*

Frozen AML samples (n=36) were retrieved from Barts Cancer Institute Biobank. Upon thawing, T cells were depleted using EasySep™ Human TCR Alpha/Beta Depletion Kit (Stem Cell Technologies). Enriched samples were plated in concentration 0.4 – 1.0 X 106/mL in Myelocult H5100 medium (Stem Cell Technologies) supplemented with 20 ng/mL IL-3, G-CSF and TPO (Biolegend) in co-culture with irradiated MS-5 cells and treated with SSI-4 (1 µM) or vehicle control with or without addition of palmitate (100) or vehicle control for 7 days. After 3 days of treatment, half of medium was exchanged with fresh medium containing the corresponding agent. After 7 days, viability was assessed using Annexin V FITC/PI stain and cell cycle was measured using PI solution. Samples were deemed sensitive to treatment if an increase in cell death greater than 5% was detected.

Samples from University Medical Center Groningen (UMCG) (n=25) were thawed and plated in co-culture with MS-5 stromal cells in Gardner’s medium with the addition of G-CSF, IL3, romiplostim, SR1 and UM171. After 2 days recovery, SSI-4 was added in two concentrations (1 and 10 µM). After 1 and 4 days of treatment, cell viability was assessed using MACSquant flow cytometer (Miltenyi Biotec), viable cells were determined as Annexin-V^-^/DAPI^-^, and area under curve (AUC) for drug sensitivity was calculated.

*In vivo experiments*

All experiments on animals were performed under UK Home Office authorisation. The mice strains used in the study were C57BL/6, NOD.Cg-*Kit^W-41J^ Tyr* ^+^ *Prkdc^scid^ Il2rg^tm1Wjl^*/ThomJ (NBSGW)^1^ and Vav-iCre^2^ and all were purchased from Jackson Laboratory. *iMLL-AF9* mice^3^ were a kind gift of Jürg Schwaller. All transgenic and knockout mice were CD45.2^+^. Congenic recipient mice were CD45.1^+^/CD45.2^+^. Mice used for support BM cells during transplantation experiments were CD45.1^+^.

For SSI-4 toxicity experiment, 13- to 15-week-old mixed gender C57BL/6 mice were treated with 10 or 30 mg/kg SSI-4 in 10% Captisol solution or vehicle control orally. All recipients were culled upon reaching treatment endpoint and their PB, spleen and BM were examined for SSI-4 effects on normal haematopoiesis. Complete blood counts and bone marrow cellularity counts were performed using Celltac α Automated Hematology Analyzer (Nihon Kohden). Only visible side effects of SSI-4 treatment were mild hair loss in animals treated with 30 mg/kg and squinting which could be prevented by application of eye drops. No changes in gross anatomy were observed upon dissection.

For syngeneic leukaemia model, CD45.1^+^/CD45.2^+^ recipient mice were lethally irradiated using a split dose of 8 Gy (two doses of 4 Gy administered at least 4 hours apart) at an average rate of 1.086 Gy/min using a RADSOURCE X-ray irradiator. 2,000 *iMLL-AF9* LSK cells were transplanted into lethally irradiated CD45.1^+^/CD45.2^+^ recipient mice together with 200,000 unfractionated support CD45.1^+^ wild-type BM cells. Engraftment and leukemic burden in PB was assessed three weeks after transplantation and animals were treated orally with 10 mg/kg SSI-4 in 10% Captisol solution or vehicle control.

*iMLL-AF9* mice were treated with doxycycline to initiate leukemic transformation. LSK cells from these mice were grown in a CFC assay in MethoCult M3434 supplemented with 250 ng/mL doxycycline for 6 days to establish their colony forming potential. 5,000 transformed leukemic cells were tail vein injected into non-irradiated 8- to 13-week-old mixed gender NBSGW mice. When leukemic burden in PB reached on average 20%, animals were randomized into treatment groups and subjected to combined chemotherapy and SSI-4 treatment. Chemotherapy was delivered in a 5 day protocol in which on days 1, 3 and 5 animals intravenously received 1.0 mg/kg doxorubicin and 50 mg/kg cytarabine in the same syringe and on days 2 and 4 animals intravenously received 50 mg/kg cytarabine. In parallel with chemotherapy, SSI-4 was delivered intraorally in the dose 10 mg/kg.

For xenotransplantation experiments, 100,000 MV-4-11 cells were tail vein injected into non-irradiated 10- to 12-week-old mixed gender NBSGW mice and began drug treatment 14 days after transplantation. Chemotherapy protocol was delivered as previously described and SSI-4 was delivered intraorally in the dose 10 mg/kg.

For patient derived xenografts (PDX), patient samples AML3 and AML5 were T-cell depleted and tail vein injected into non-irradiated 8- to 10-week-old mixed gender NBSGW mice (2 and 4 million cells per mouse, respectively). Engraftment of human hematopoietic cells was confirmed at weeks 8 and 10 by BM sampling and testing for the presence of human CD45^+^ cells. Upon engraftment confirmation, mice were randomized per treatment group and orally treated with 10 mg/kg SSI-4 in 10% Captisol solution or vehicle control. To allow for comparison between different patient samples, engraftment in each animal was normalized to the average engraftment of that patient sample

For doxycycline (Dox) treatment needed for leukemic transformation of *iMLL-AF9* model, mice were provided *ad libitum* access to drinking water containing 2 mg/mL DOX with 30% sucrose. All animals were culled upon reaching either treatment endpoint or their humane endpoint as recorded in survival curves.

*Leukemic transformation*

*Meis1/Hoxa9* transformed murine leukemic cells and *iMLL-AF9* murine leukemic cells were grown in IMDM supplemented with 10% FBS and 10 ng/ml SCF, 5 ng/ml IL-3 and 5 ng/ml IL-6 or 20 ng/ml SCF, 10 ng/ml IL-3, 10 ng/ml IL-6 and 250 ng/mL doxycycline respectively.

*Proliferation rate assays*

To measure cell line proliferation rate, cells were plated at a starting concentration 300 000 cell/mL and treated with SSI-4 (1 µM). Every 72h, viable cell counts were determined using trypan blue exclusion, cell were washed in fresh medium, their concentration readjusted to 300 000 cell/mL and fresh SSI-4 (1 µM) was added. This was repeated for 6 cycles of 72h with full treatment duration of 21 days. Cumulative number of cell division was calculated for each timepoint.

To measure proliferation rate of CRISPR KO cells, a competition assay was used. 100 000 Cas9/KO-BFP non-targeting (NT) gRNA, SCD gRNA 1 or 2 per ml were mixed with 100 000 Cas9 cells with no BFP (parental) and plated at a final concentration 200 000 cell/mL. At days 3 and 5 the ratio between BFP positive and negative cells were measured by flow cytometry and data were normalized to NT group.

*Flow cytometry*

Cell viability and cell death induction were determined using FITC-conjugated anti-Annexin V antibody with propidium iodide or Zombie NIR™ (BioLegend) stains or PE-conjugated anti-Annexin V antibody with 7-AAD stain. Double negative cells were deemed viable. Cell cycle progression was determined using PI solution (50 μg/ml PI, 10 mm Tris, pH 8.0, 10 mm NaCl, 10 μg/ml RNase A, 0.1% Igepal) and percentage of cells in each cell cycle phase was calculated using Dean Jett Fox model.

For lipid peroxidation measurement cells were incubated with 4 µM Bodipy 581/591 for 30 min at 37 °C with gentle shaking, protected from light. Cells were washed twice with PBS and analysed by flow cytometry. Lipid peroxidation was determined as the ratio of MFI in the green channel (530) vs MFI in the red channel (610) per manufacturer’s instructions.

For lipid uptake measurement cells were incubated with 1 µM C1-Bodipy C12 500/510 at 37 °C and 5% CO_2_ for 24 h. Cells were washed twice with PBS and analysed by flow cytometry.

BM cells were isolated by crushing tibias and femurs using a pestle and mortar. Splenic cells were prepared by mashing the tissue and passing through a 70 µm strainer. Erythrocytes in PB were lysed using ammonium chloride solution. Single cell suspensions from BM, spleen or PB were incubated with Fc block and then stained with antibodies. For HSC and progenitor cell analyses, unfractionated BM cells were stained with lineage markers containing biotin-conjugated anti-CD4, anti-CD5, anti-CD8a, anti-CD11b, anti-B220, anti-Gr-1 and anti-Ter119 antibodies together with BV711-conjugated anti-c-Kit, APC-Cy7-conjugated anti-Sca-1, PE-conjugated anti-CD48 and PE-Cy7-conjugated anti-CD150 antibodies. Biotin-conjugated antibodies were then stained with PB-conjugated streptavidin. For analyses of differentiated cells, PB was stained with PerCP-conjugated anti-B220 and APC-Cy7-conjugated anti-CD19 antibodies for B cells; APC-conjugated anti-CD11b and PE-Cy7-conjugated anti-Gr-1 for myeloid cells; PE-conjugated anti-CD4 and anti-CD8 antibodies for T cells.

To distinguish CD45.2^+^-donor derived cells in PB or BM of transplanted mice, BV711-conjugated anti-CD45.1 and FITC-conjugated anti-CD45.2 antibodies were used, and to distinguish human versus mouse CD45^+^ positive cells PB-conjugated anti-human CD45 and APC-conjugated anti-mouse CD45 antibodies were used. TO-PRO-3 was used for dead cell exclusion. Human myeloid cells in xenograft models were distinguished using PE-conjugated anti-human CD33 and B-cell exclusion was done using BV711-conjugated anti-human CD19.

Flow cytometry analyses were performed using LSRFortessa and FACSymphony A3 (BD) instruments and all data analysis was performed using FlowJo 10.0 software.

*Lentiviral transduction*

293T-Phoenix cells were transfected with pMD2.G, psPAX2 and construct plasmid using TransIT (Mirus Bio) for transient transfection. After overnight incubation at 37 °C with 5% CO2, media containing virus was harvested and filtered through a 0.45 µm filter to remove any cells. For transduction of cell lines, 1.8 million cells were spinoculated with virus containing medium and 4 µg/ml polybrene (Santa Cruz Biotechnology, TR1003) for 90 min at 900 × g. After 24 h, free viral particles removed by washing three times with sterile PBS and cells were subjected to 2 µg/mL puromycin selection for 14 days.

*Generation of CRISPR knockout clones*

The knock-out of genes was accomplished using the CRISPR/Cas9 system. For this MV411 and THP-1 cell lines were transduced with Cas9 lentivirus (Addgene, lentiCas9-Blast), generating Cas9 expressing cell lines. Functional gRNA sequences (Supplemental data 9) were obtained from genome-wide gRNA library and ligated in the backbone, conjugating SCD gRNA with BFP (Addgene, pKLV2-U6gRNA5(BbsI)-PGKpuro2ABFP-W). Virus for Cas9, SCD gRNA and NT gRNA were produced and transduced as described above. Successfully transfected cells were identified by puromycin selection. Obtaining single cell colonies was performed using methylcellulose media (H4531; STEMCELL Technologies, Cambridge, UK). Genetically modified cell lines were expanded from selected single cell colonies in RPMI medium for two weeks and SCD expression was validated using western blot.

*Western blotting*

Cells were lysed using cell lysis buffer (Cell Signaling Technology) supplemented with protease and phosphatase inhibitors (Sigma-Aldrich, Millipore). DNA was sheared by passing the sample through a fine gauge needle 8 times before centrifugation for 10 min at 14,000 x g. The protein extracts were subjected to SDS–PAGE (Invitrogen) and then transferred onto PVDF membranes (Sigma-Aldrich) using wet transfer. Membranes were blocked with 5% milk powder in 1x PBS-T for 30 mins then incubated overnight at 4 °C with primary antibodies. After 2h incubation at RT with appropriate horseradish peroxidase-coupled secondary antibody, bands were detected with enhanced chemiluminescence substrates (BioRad, ThermoFisher) and visualized using an Amersham Imager (GE Healthcare) or a ChemiDoc Imaging system (BioRad).

For analysis of phosphorylated vs total proteins equal aliquots of protein solution were loaded to parallel gels and processed simultaneously. Band intensity quantification (densitometry) was performed using Image J software and normalized to ß-actin. Antibodies used are enclosed in Supplemental data 9.

*Real Time quantitative PCR*

The RNA was isolated using Direct-zol RNA Miniprep kit (Zymo Research) following the manufacturer’s protocol and reverse transcribed using High-Capacity cDNA Reverse Transcription Kit (Applied Biosystems). For real-time quantitative PCR (qPCR), 5 ng of cDNA, 5 µL of PowerUp SYBR Green MasterMix (Applied Biosystems) and 2 pmol of primers (Suppl. data 10) were used per well of 384-well plate. Reactions were performed in triplicate using C1000 Thermocycler 384well (BioRad). Gene expression was quantified using comparative ΔΔ-Ct method and *ACTB* was used as the housekeeping gene. Data is expressed as log2 fold change in comparison to control sample and represents results of three independent experiments measured in duplicate.

*RNA sequencing and analysis*

RNA Sequencing was provided by Novogene UK Company Limited (Cambridge, UK). The RNA was isolated using Direct-zol RNA Miniprep kit (Zymo Research) following the manufacturer’s protocol. Messenger RNA was purified from total RNA using poly-T oligo-attached magnetic beads (poly-A enrichment). Generated library was sequenced on an Illumina platform and paired-end reads were generated.

Reads were mapped to a reference genome using Hisat2 and Featurecounts was used to count the read numbers mapped of each gene. Differential expression analysis of two conditions was performed using the edgeR R package. Corrected p-value of 0.005 and |log2(Fold Change)| of 1 were set as the threshold for significantly differential expression. Enrichment analysis based on KEGG pathways (<http://www.genome.jp/kegg/>) was performed using clusterProfiler R package or Enrichr webtool (<https://maayanlab.cloud/Enrichr/>). Gene set enrichment analyses (GSEA) and single sample GSEA (ssGSEA) were performed using the local version of the GSEA analysis tool (<http://www>.broadinstitute.org/gsea/index.jsp). For GSEA analysis normalized read counts for all conditions were used and genes were ranked using the signal-to-noise metric and FDR and NES were calculated using 1000 gene-set permutation.

*Glucose labelling*

Cells were grown for 24h in RPMI medium with no glucose, supplemented with 10% FBS, 50 IU/ml penicillin and 50 μg/ml streptomycin and 2 g/L U-¹³C_16_-Glucose (CK Isotopes) and treated with SSI-4 (1 µM) with or without the addition of BSA-conjugated sodium oleate (100 µM) or sodium palmitate (100 µM). At the end of the treatment, cells were counted in triplicates for normalization, washed in cold PBS and apolar fraction of cell pellets was isolated using methanol:chloroform extraction.

In analysis, fatty acids containing isotope ¹³C peaks m+0 and m+1 were marked as unlabelled and the ones containing m+2 and higher as labelled.

*Metabolomics experiments*

For lipidomics analysis, lipid species were extracted from cell pellets using monophasic isopropanol extraction and analysed using liquid chromatography and a Q Exactive™ Hybrid Quadrupole-Orbitrap™ Mass Spectrometer (ThermoFisher)^4^. Peak detection, alignment and deconvolution was performed with Compound Discoverer software (ThermoFisher) and lipid annotation was performed with LipiDex software using the parameters used in the original publication^5^. Additional analysis of the lipidomics dataset was performed with the LipidSuite webtool (<https://suite>.lipidr.org).

For fatty acid profiling, apolar metabolites were isolated from cells using chloroform:methanol extraction (2:1, v/v, both HPLC grade, Fisher) and fatty acids partitioned from polar metabolites by resuspension of dried extracts in chloroform:methanol:water (1:3:3, v/v, HPLC grade, Fisher). Data acquisition was performed using an Agilent 7890B-7000C GC-triple-quadrupole MS in EI mode after derivatization of twice methanol-washed dried lower (apolar) phase by addition of 25 μL chloroform/methanol (2:1, v/v) and 5 μL tetramethylammonium hydroxide (TMAH, RT, no incubation) for fatty acids. Use of TMAH derivativisation allowed for extraction of both free fatty acids and fatty acid bound in lipid fractions. GC-MS parameters were as follows: carrier gas, helium; flow rate, 0.9 mL/min; column, DB-5MS (Agilent); inlet, 250°C; temperature gradient, 70°C (1 min), ramp to 230°C (15°C/min, 2 min hold), ramp to 325°C (25°C/min, 3 min hold). Scan range was m/z 50-565. Data was acquired using MassHunter software (version B.07.02.1938). Data analysis was performed using MANIC software, an in house-developed adaptation of the GAVIN package^6^. Fatty acids were identified and quantified by comparison to authentic standards and ^13^C_1_-lauric acid as an internal standard (Cambridge Isotope Laboratories) (run in the same sample batch). Label incorporation was calculated as the percentage of the metabolite pool containing one or more ^13^C atoms after correction for natural abundance. The abundance of each FA was estimated by calculation of the amount in the sample (relative to an internal standard) compared to a known amount of an authentic standard (relative to the same internal standard).

STATISTICS AND REPRODUCIBILITY

All data was analysed and visualised in Prism 9.0 and 10.0 (GraphPad) and all data are shown as mean ± standard error of the mean, unless otherwise stated. The cohorts were dichotomized into groups with high and low gene expression after calculating the optimal cut point value using the receiver operating characteristic (ROC) curve for censored overall survival data. Overall survival was plotted using Kaplan–Meier plots, using Cox proportional hazard regression to compare the differences between the curves, providing the Hazard ratio (HR) and the 95% confidence interval (CI). According to data availability, we adjusted prognosis prediction for confounders as follows: age (as continuous variable), sex (male vs. female), white blood cell counts (WBC, as continuous), and European LeukemiaNet categorization (ELN2010 or ELN2017). The difference between multiple experimental groups was analysed by two-way ANOVA (Kruskal–Wallis test, post hoc Dunn analysis) or two-tailed paired or unpaired Student’s t test. IC50 and cell viability curves were determined using non-linear regression analyses. Correlation was calculated using Pearson or Spearman correlation coefficients. P-values are indicated in Figure legends.

**Supplemental references:**

1. McIntosh Brian E, Brown Matthew E, Duffin Bret M, Maufort John P, Vereide David T, Slukvin Igor I*, et al.* Nonirradiated NOD,B6.SCID Il2rγ−/− KitW41/W41 (NBSGW) Mice Support Multilineage Engraftment of Human Hematopoietic Cells. *Stem Cell Reports* 2015 2015/02/10/; **4**(2)**:** 171-180.

2. de Boer J, Williams A, Skavdis G, Harker N, Coles M, Tolaini M*, et al.* Transgenic mice with hematopoietic and lymphoid specific expression of Cre. *European Journal of Immunology* 2003 2003/02/01; **33**(2)**:** 314-325.

3. Stavropoulou V, Kaspar S, Brault L, Sanders Mathijs A, Juge S, Morettini S*, et al.* MLL-AF9 Expression in Hematopoietic Stem Cells Drives a Highly Invasive AML Expressing EMT-Related Genes Linked to Poor Outcome. *Cancer Cell* 2016 2016/07/11/; **30**(1)**:** 43-58.

4. Blomme A FC, Mui E, Patel R, Ntala C, Jamieson LE, Planque M, McGregor GH, Peixoto P, Hervouet E, Nixon C, Salji M, Gaughan L, Markert E, Repiscak P, Sumpton D, Blanco GR, Lilla S, Kamphorst JJ, Graham D, Faulds K, MacKay GM, Fendt SM, Zanivan S, Leung HY. 2,4-dienoyl-CoA reductase regulates lipid homeostasis in treatment-resistant prostate cancer *Nature Communications* 2022; **11:** 2508.

5. Hutchins PD RJ, Coon JJ. LipiDex: An Integrated Software Package for High-Confidence Lipid Identification *Cell Systems* 2018; **6:** 621-625.e625.

6. Behrends V, Tredwell GD, Bundy JG. A software complement to AMDIS for processing GC-MS metabolomic data. *Analytical Biochemistry* 2011 2011/08/15/; **415**(2)**:** 206-208.

**
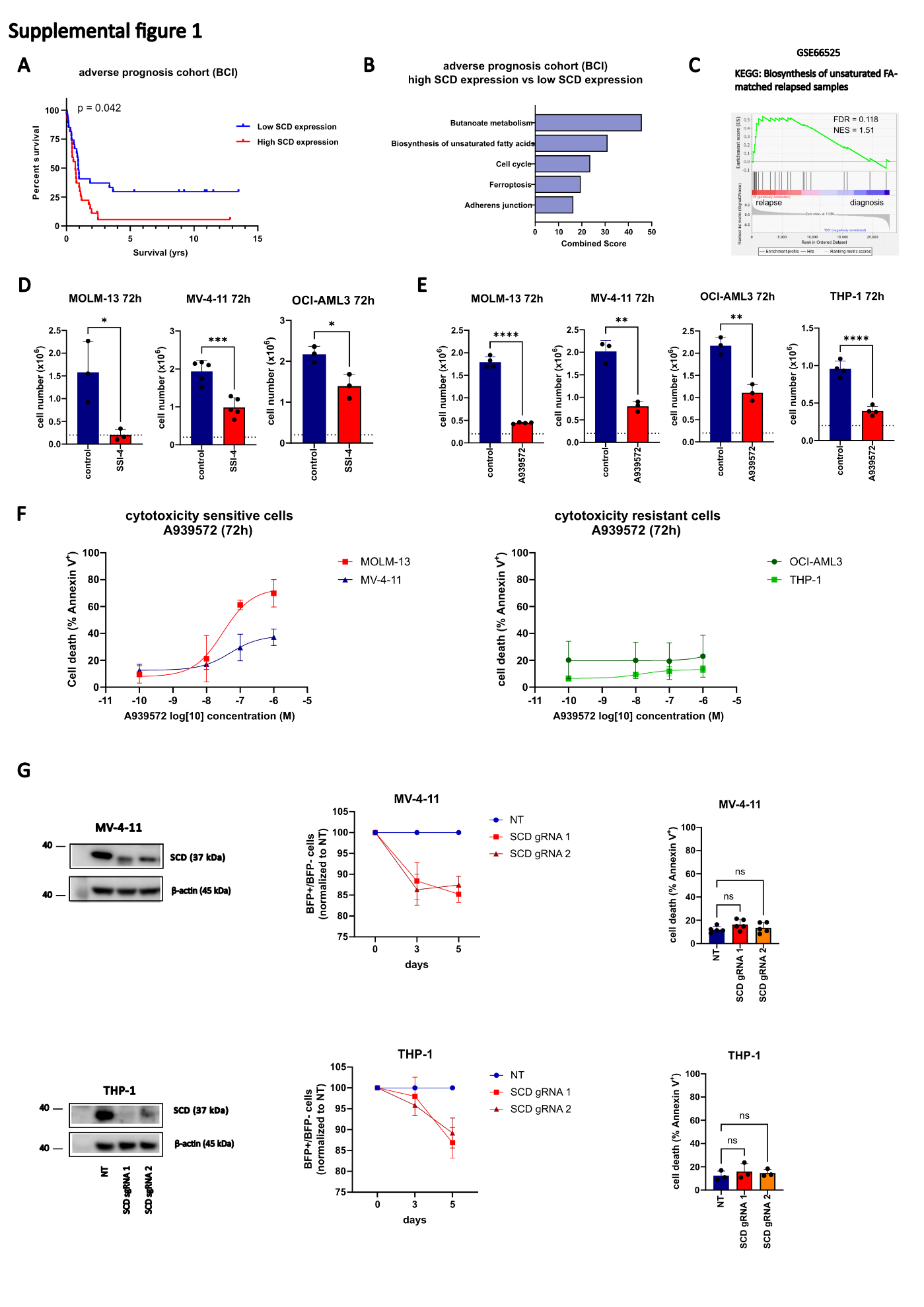
Supplemental Fig. 1. Increased unsaturated fatty acids biosynthesis correlates with worse prognosis in AML. Sensitivity to SSI-4 is phenocopied by other means of SCD inhibition.**

(A) Kaplan-Meier curve comparing overall survival in BCI AML patients cohort with adverse prognosis after ELN (n=54) dichotomized using median SCD expression. Log rank (Mantel-Cox) test was used for determining significance. (B) Significantly enriched KEGG signatures in patients with highest SCD expression (n=10) compared with patients with lowest *SCD* expression (n=10) in BCI AML patients cohort with adverse prognosis after ELN (n=54) ranked by combined score from Enrichr enrichment analysis. (C) Gene set enrichment analysis (GSEA) for KEGG pathway Biosynthesis of unsaturated fatty acids in paired relapse-diagnosis samples (GSE66525). (D) Cell counts for MOLM-13, MV-4-11 and OCI-AML3 treated with SSI-4 (1 µM) for 72h. Dotted line represents initial plating concentration of 200 000 cells per mL. (E) Cell counts for MOLM-13, MV-4-11 and OCI-AML3 treated with A939572 (10 µM) for 72h. Dotted line represents initial plating concentration of 200 000 cells per mL. (F) MOLM-13, MV-4-11, OCI-AML3, THP-1 and HL-60 cells were treated with the SCD inhibitor A939572 (10 nM, 100 nM, 1 µM) or corresponding vehicle for 72h. Dying cells were recognized by expression of Annexin V. Cells with less than 10% increase in cell death were designated to the resistant group. Results are presented as non-linear regression. (G) Representative western blot (n=3) confirming SCD downregulation in MV-4-11 and THP-1 cells transduced with NT gRNA, SCD gRNA1 and SCD gRNA2 (left panels). Competition growth assays measuring the ratio of BFP -positive (gRNA expressing cells) to BFP-negative cells (wild type) after 3 and 5 days in MV-4-11 and THP-1 cell lines (middle panels). Percentage of dying cells in baseline normoxic conditions in MV-4-11 and THP-1 cell lines with SCD downregulation (right panel). Data are mean ± SD. * p < 0.05, ** p < 0.01, *** p < 0.001, **** p < 0.0001.

**
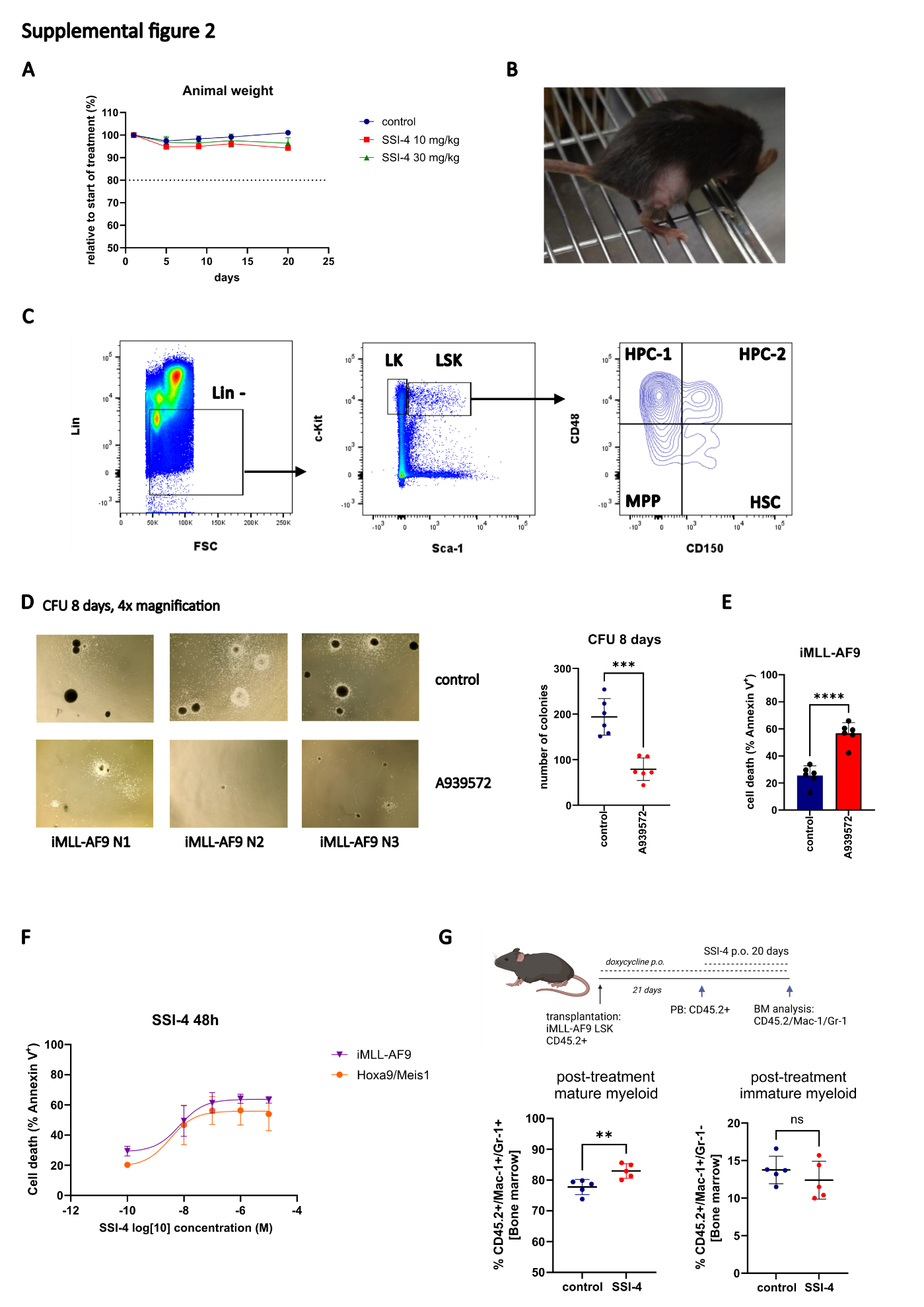
Supplemental Fig. 2. AML models sensitivity to SSI-4 *in vivo* demonstrates comparable variability of response to the one observed *in vitro*.**

(A) Weight change in animals treated with SSI-4 (10 and 30 mg/kg) for 3x5 days. (B) Visual representation of hair loss observed in animals treated with 30 mg/kg SSI-4. (C) Gating strategy for hematopoietic progenitors. (D) Colony forming units assay of leukemic transformed LSK cells from three mice (N1-N3) expressing *iMLL-AF9* treated with A939572 (1 µM). (E) Percentage of Annexin V expressing *iMLL-AF9* cells after 48h of A939572 (1 µM) in liquid culture. (F) Leukemic *Hoxa9/Meis1* and *iMLL-AF9* cells were treated with SSI-4 (0.01-10 µM) or corresponding vehicle. Dying cells were determined as Annexin-V^+^ (G) CD45.2^+^ LSK cells from *iMLL-AF9* mice were transplanted to lethally irradiated syngeneic CD45.1^+^/CD45.2^+^ recipient mice (n=10). After engraftment was confirmed in PB, animals were treated with 10 mg/kg SSI-4 or corresponding vehicle orally for 20 days. Percentage of CD45.2^+^/Mac-1^+^/Gr-1^+^ and CD45.2^+^/Mac-1^+^/Gr-1^-^ cells in the BM at the end of the experiment. Data are mean ± SD. * p < 0.05, ** p < 0.01, *** p < 0.001, **** p < 0.0001.

**
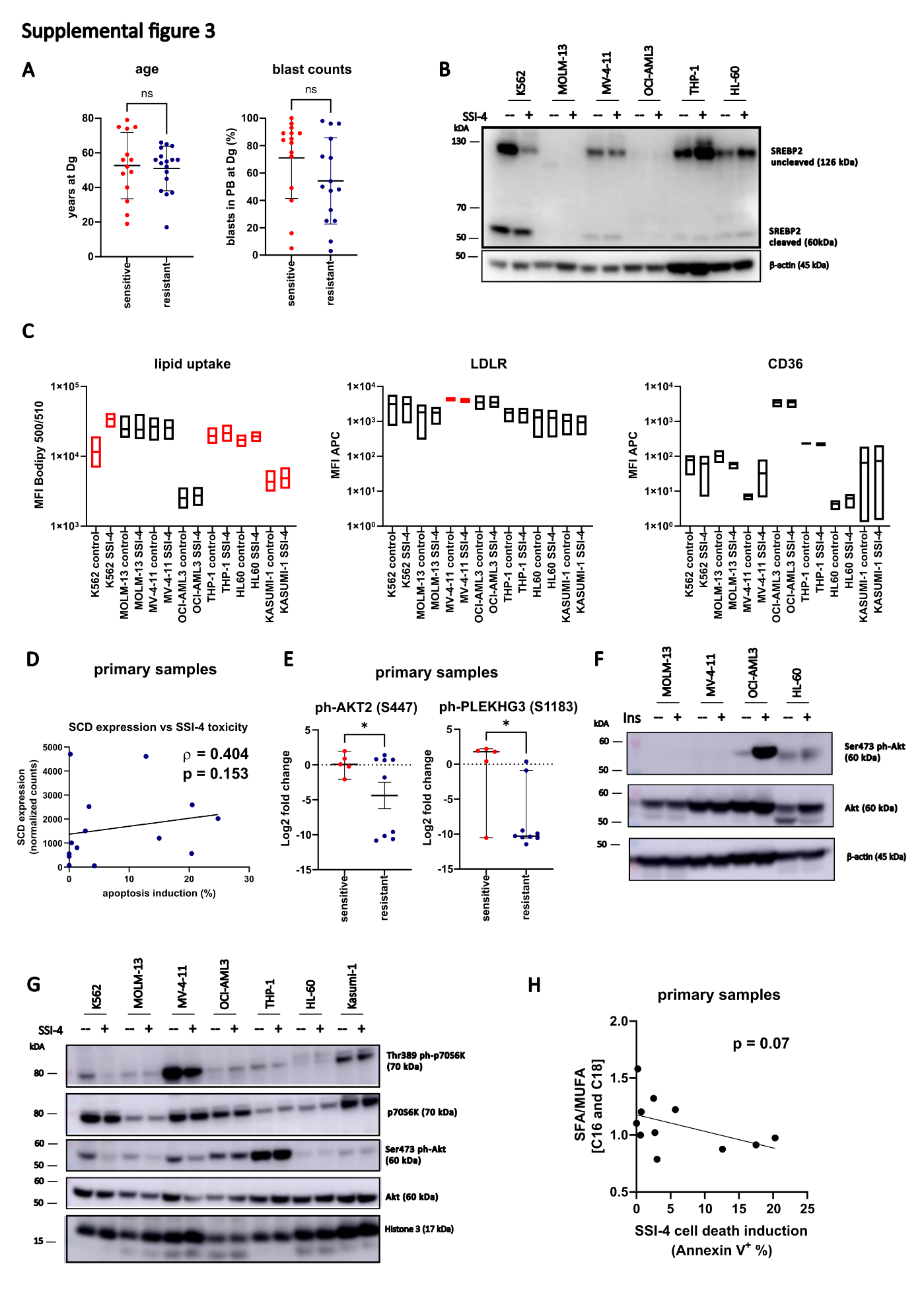
Supplemental Fig. 3. Sensitivity to SSI-4 in AML cells is neither associated with specific mutations nor with the levels of uptake of external lipids.**

(A) Age and blast count distribution in SSI-4 sensitive and resistant group of patient samples. (B) representative western blot (n=2) of SREBP2 expression in AML cell lines treated with SSI-4 (1 µM). (C) K562, MOLM-13, MV-4-11, OCI-AML3, THP-1, HL-60 and Kasumi-1 cells were treated with SSI-4 (1 µM) and lipid uptake was measured using Bodipy 500/510 stain as well as the expression of LDLR and CD36. Red bars present samples where difference between SSI-4 treated and control cells was statistically significant. (D) Correlation of SCD expression and induction of apoptosis in response to SSI-4 in primary AML samples (n=14). (E) Phosphoproteomic analysis for phosphorylated AKT2 and PLEKHG3 in 5 sensitive and 9 resistant AML patients from BCI Adverse prognosis cohort expressed as log2 fold change towards average target relative intensity in analysis. Data are mean ± 95% confidence interval. (F) Representative western blot (n=3) of changes in Akt phosphorylation in response to 10 minutes insulin (10 µg/mL) pulse after 24h of serum starvation in SCDi sensitive (MOLM-13, MV-4-11) and resistant (OCI-AML3, HL-60) cell lines. (G) Representative western blot (n=2) of changes in Akt and p70S6K phosphorylation in response to SSI-4 (1 µM) treatment in cell lines tested. (H) Correlation plot presents association between SFA/MUFA ratio and cell death induction as measured by Annexin-V expression in response to SSI-4 treatment in primary samples (n=11). Data are mean ± SD. * p < 0.05, ** p < 0.01, *** p < 0.001, **** p < 0.0001.

**
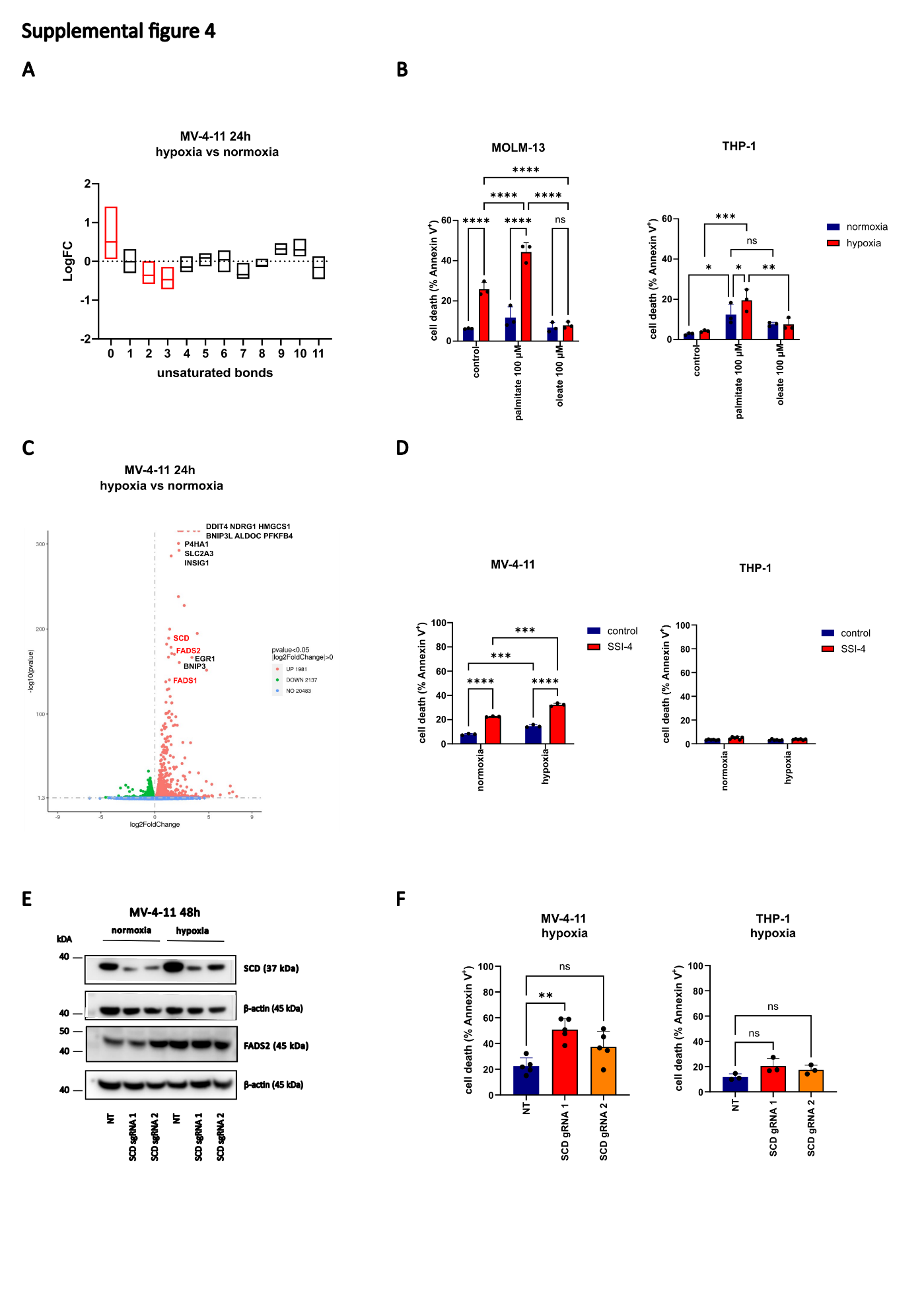
Supplemental Fig. 4. Increased dependency on SCD in hypoxia sensitizes cells to SSI-4.**

(A) Lipidomics analysis on MV-4-11 cells after 24h in hypoxic conditions (3% O_2_). Graph represents enrichment analysis per number of desaturated bonds of cells in hypoxia in comparison to control (Q1-Q3 with line at median value) with statistically significant groups marked in red. (B) Cell death induction in SCDi sensitive MOLM-13 and SCDi resistant THP-1 cells after 72h in hypoxia with or without addition of oleate (100 µM) or palmitate (100 µM). (C) RNA sequencing results for MV-4-11 cells after 24h in hypoxic conditions. Fatty acid desaturases are presented in red. (D) Cell death induction MV-4-11 cells after 72h in hypoxia in combination with SCD inhibition using SSI-4 (1 µM) or A939572 (1 µM). (E) Representative western blot (n=3) showing the response to 48h of hypoxia in MV-4-11 cells with downregulated SCD. (F) Percentage of Annexin V expressing MV-4-11 and THP-1 with downregulated SCD after 72h of or hypoxic conditions. Data are mean ± SD.* p < 0.05, ** p < 0.01, *** p < 0.001, **** p < 0.0001.

**
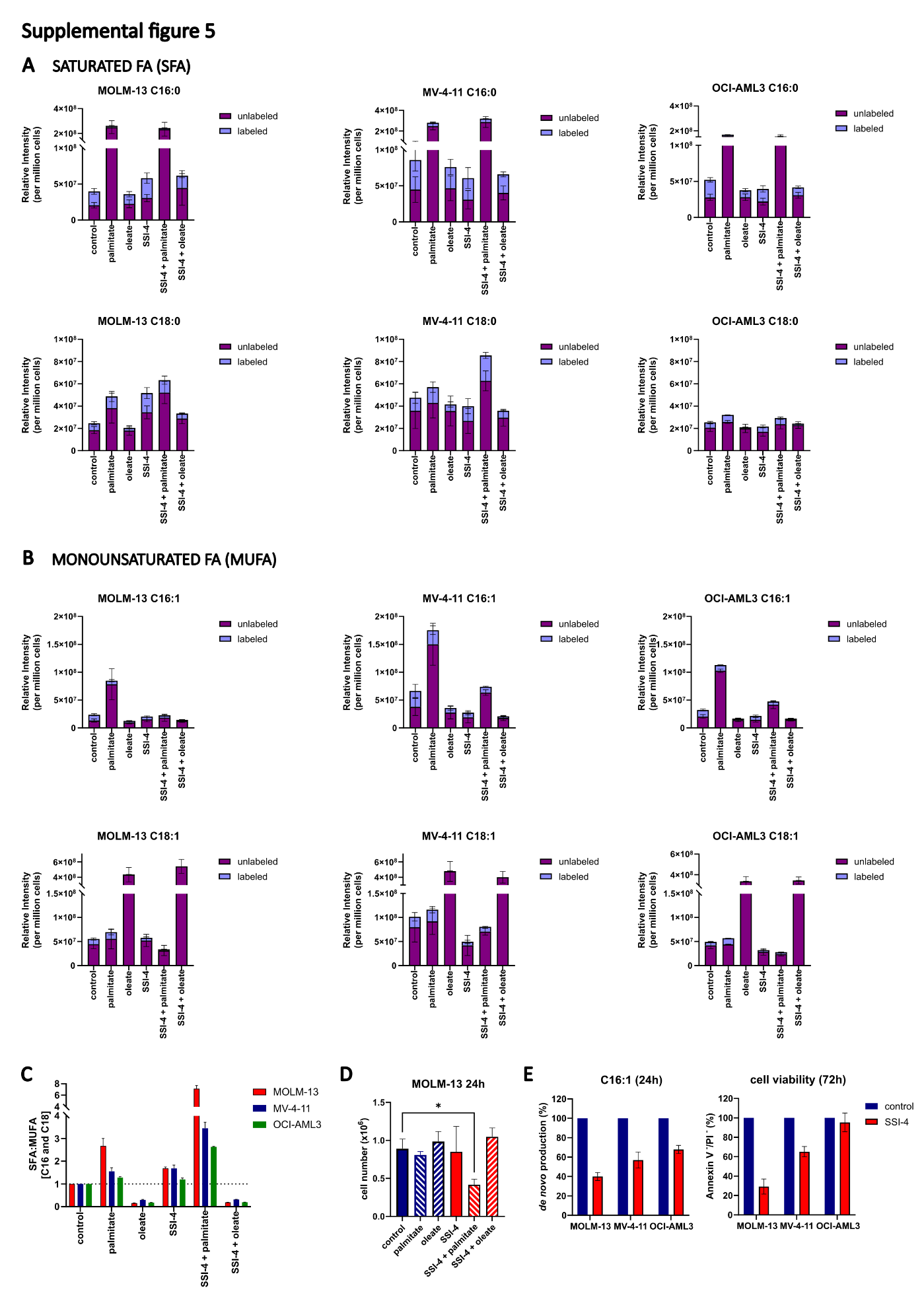
Supplemental Fig. 5. Fatty acids profiles in SSI-4 sensitive and resistant cells in the presence of oleate and palmitate.**

(A) Levels of ^13^C-glucose labeled (m+2 and higher) and unlabeled (m+0, m+1) saturated fatty acids (SFA) palmitate (C16:0) and stearate (C18:0) in MOLM-13, MV-4-11 and OCI-AML3 cells after 24h of SSI-4 (1 µM) treatment with or without the addition of oleate (100 µM) or palmitate (100 µM). (B) Levels of ^13^C-glucose labeled (m+2 and higher) and unlabeled (m+0, m+1) monounsaturated fatty acids (MUFA) palmitoleate (C16:1) and oleate (C18:1) in MOLM-13, MV-4-11 and OCI-AML3 cells after 24h of SSI-4 (1 µM) treatment with or without the addition of oleate (100 µM) or palmitate (100 µM). (C) Ratio of C16 and C18 SFA and MUFA normalized to control in corresponding experimental conditions. (D) Cell counts of MOLM-13 cells after 24h of respective treatment conditions. (E) Drop in C16:1 production in MOLM-13, MV-4-11 and OCI-AML3 cells in response to SSI-4 (1 µM) treatment for 24h paralleled to a drop in cell viability after 72h of respective treatment. Viable cells were determined as Annexin V^-^/PI^-^ and all data are normalized to control conditions. All experiments are done in three independent replicates (n=3). Data are mean ± SD. * p < 0.05, ** p < 0.01, *** p < 0.001, **** p < 0.0001.

**
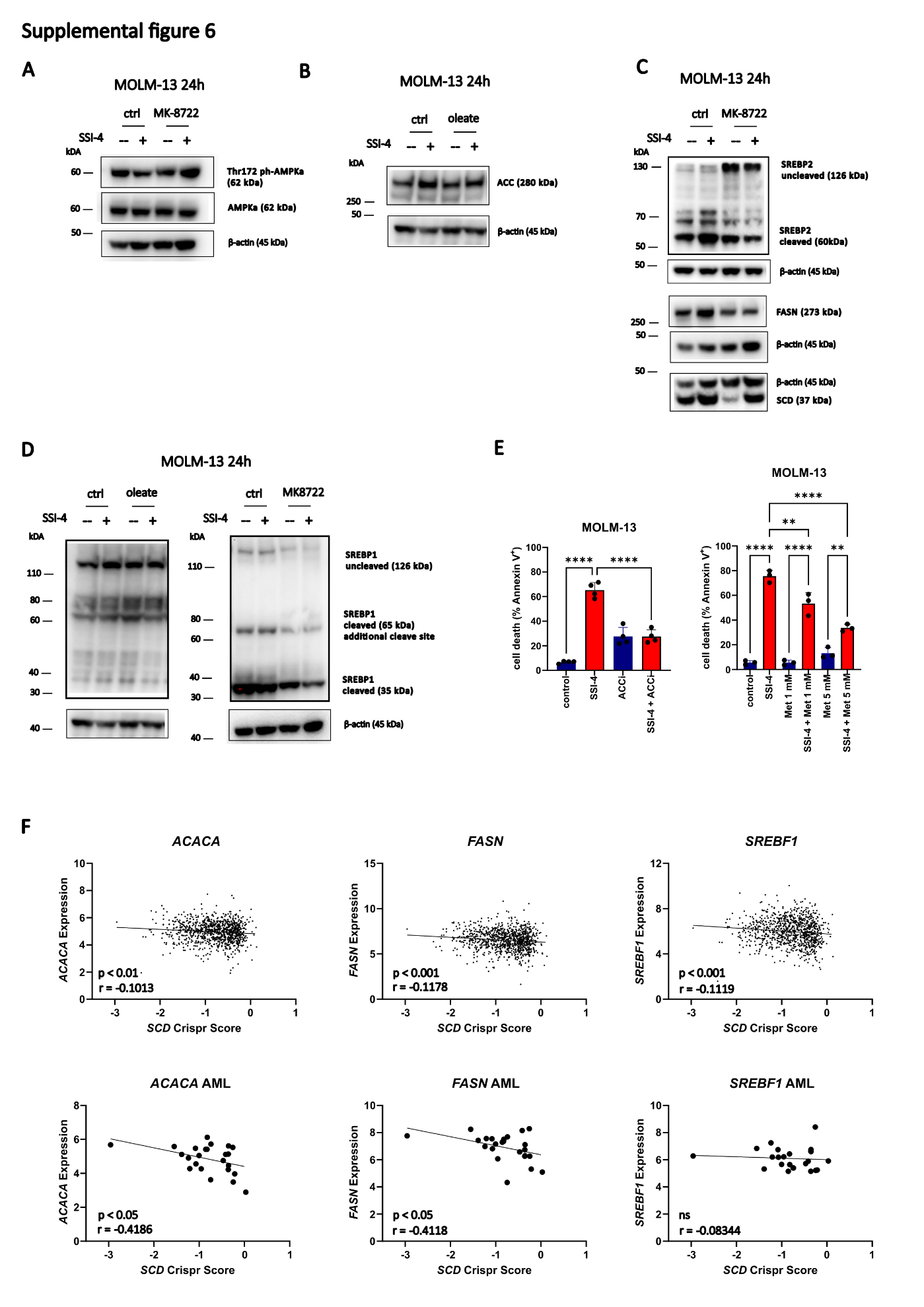
Supplemental Fig. 6. Functional inhibition of *de novo* FA synthesis pathway rescues SSI-4 mediated toxicity.**

(A-D) Representative western blots (n=3) of MOLM-13 cells treated for 24h with SSI-4 (1 µM) or vehicle control with or without addition of oleate (100 µM) or direct AMPK activator MK-8722 (10 µM). (E) MOLM-13 cells were treated for 72h with SSI-4 (1 µM) with or without addition of FASN inhibitor Fasnall (20 µM), ACC inhibitor PF-05221304 (5 µM), MK-8722 (10 µM) or AMPK activator metformin (1 and 5 mM). Dying cells were determined as Annexin-V^+^. (F) DepMap dataset was analyzed for correlation (Pearson r) between SCD Crispr scores and expression levels of *ACACA (*coding for ACC*)* and *FASN.* Results on all cancer cell lines in dataset are presented in upper panels and results on AML cell lines in lower panels. Data are mean ± SD.* p < 0.05, ** p < 0.01, *** p < 0.001, **** p < 0.0001.

**
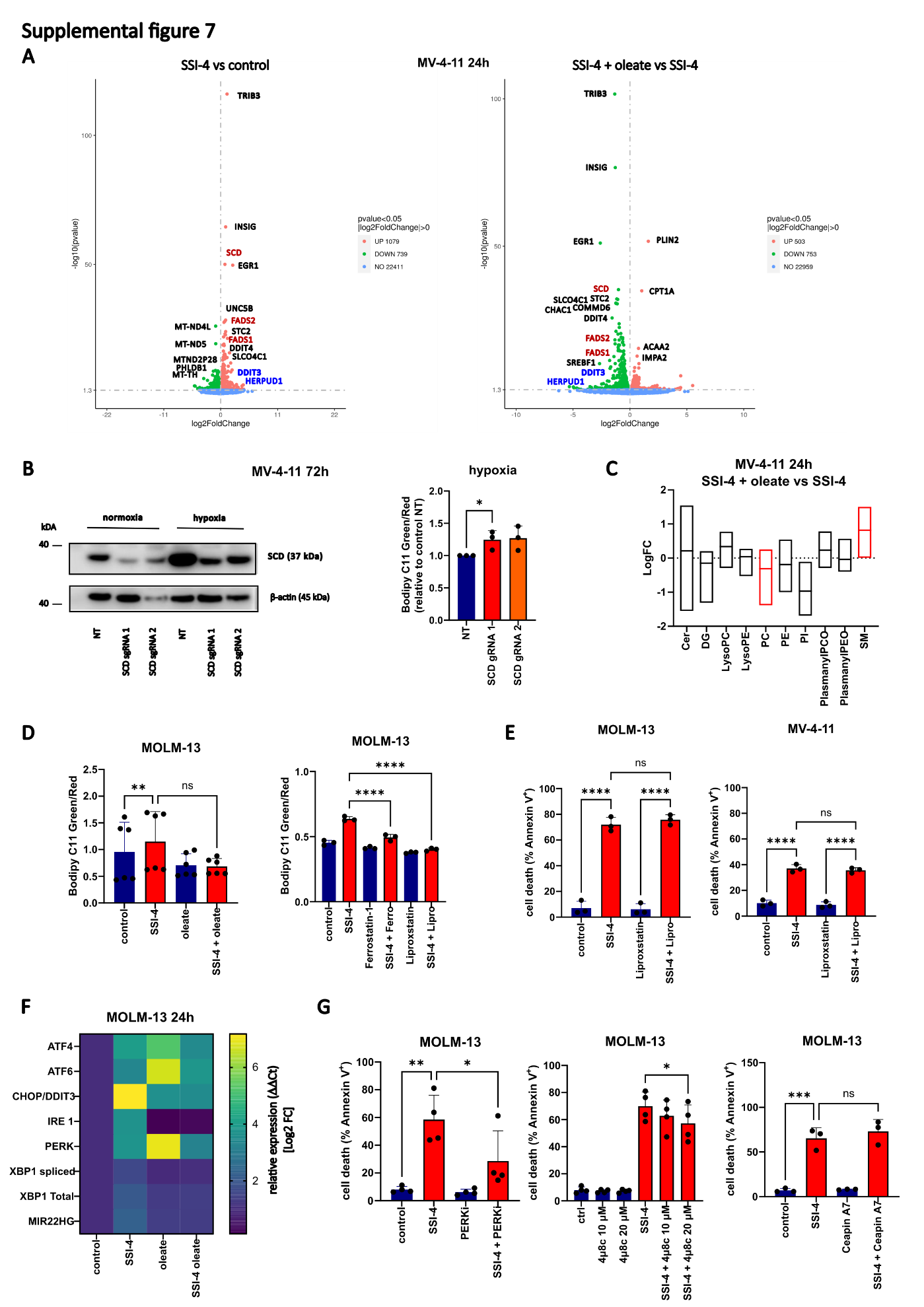
Supplemental Fig. 7. SSI-4 induces both lipid peroxidation and broad activation of ER stress response.**

(A) RNA sequencing results for MV-4-11 cells treated with SSI-4 (1 µM) for 24h with (right panel) or without the presence of oleate (100 µM) (left panel). Most differentially expressed genes are highlighted. Fatty acid desaturases are presented in red and ER stress-related genes in blue. (B) Lipid peroxidation measured by Bodipy C11 in MV-4-11 cells with downregulated SCD after 72h in hypoxic conditions (3% O_2_). Representative western blot (n=3) demonstrating SCD levels in normoxic and hypoxic conditions is shown. (C) Lipidomics analysis on MV-4-11 cells treated with SSI-4 (1 µM) for 24h with the presence of oleate (100 µM). Graph represents enrichment analysis per lipid groups of SSI-4 + oleate treated cells in comparison to oleate alone (Q1-Q3 with line at median value) with statistically significant lipid groups marked in red. (D) Rescue of lipid peroxidation induction in response to SSI-4 (1 µM, 24h) using oleate (100 µM), as well as lipid peroxidation inhibitors ferrostatin-1 (5 µM) and liproxstatin (2 µM) in MOLM-13 cells. Lipid peroxidation was measured was measured using Bodipy C11. (E) MOLM-13 and MV-4-11 cells were treated for 72h with SSI-4 (1 µM) or vehicle control with or without addition of Liproxstatin (2 µM). (F) MOLM-13 were treated for 24h with SSI-4 (1 µM) with or without addition of oleate (100 µM) and expression of ER Stress related genes was determined using qPCR. Heatmap represents Log_2_ fold changes of genes measured normalized to ß-actin and control sample (2^-ΔΔCt^). (G) MOLM-13 cells were treated for 72h with SSI-4 (1 µM) with or without addition of PERK inhibitor GSK2656157 (5 µM) IRE-1 inhibitor 4µ8c (10 and 20 µM) and ATF6 inhibitor Ceapin A7 (5 µM) Cell death was determined by Annexin-V expression. Data are mean ± SD * p < 0.05, ** p < 0.01, *** p < 0.001, **** p < 0.0001.

**
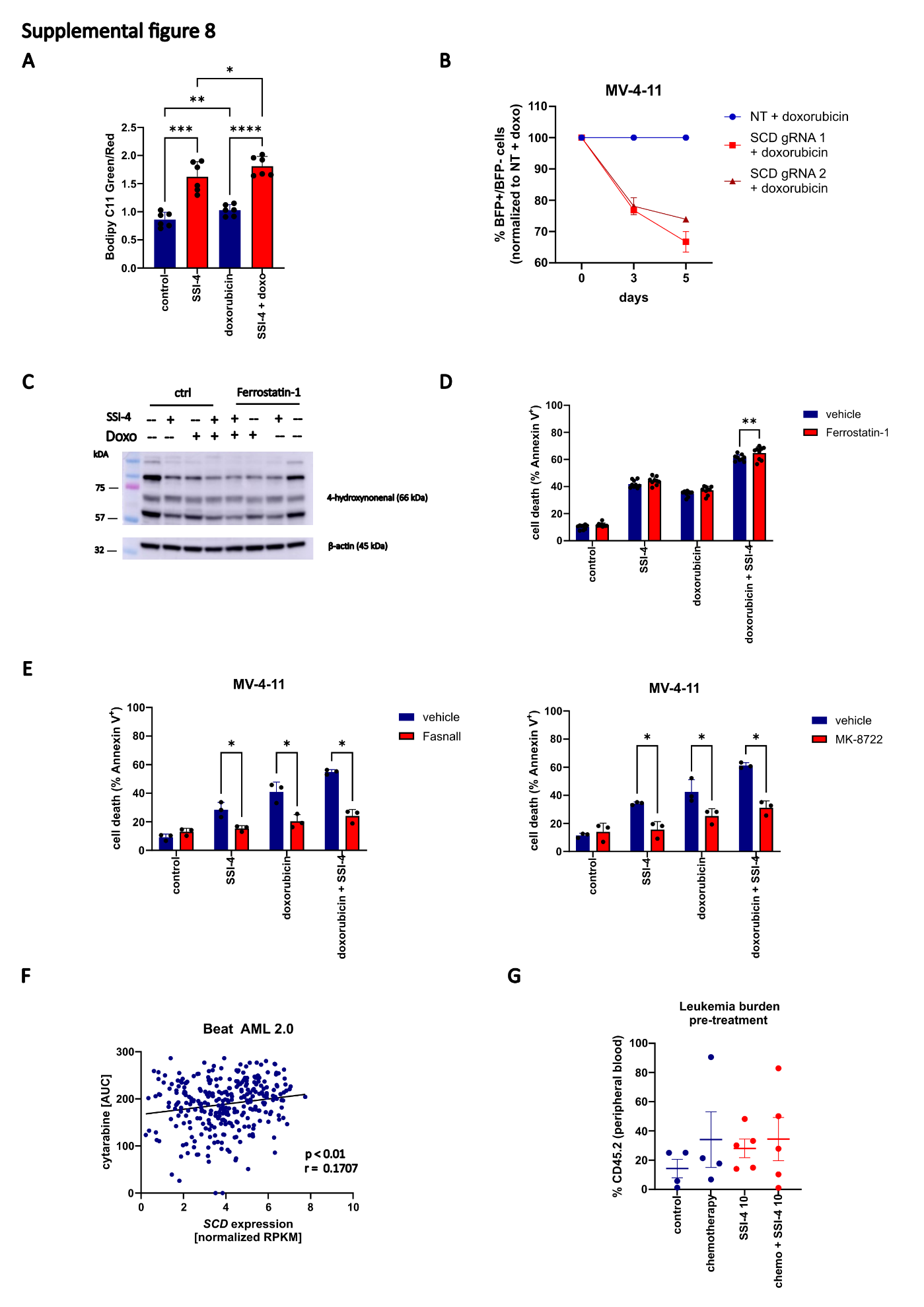
Supplemental Fig. 8. Lipotoxicity enhances the anti-leukemic effects of doxorubicin.**

(A) Lipid peroxidation in MV-4-11 cells treated for 72h with SSI-4 (1 µM) with or without addition of doxorubicin (1 µM) was measured using Bodipy C11. (B) Competition growth assay between BFP^+^ and BFP^-^ MV-4-11 cells (MV-4-11 WT *Cas9* expressing) for cells expressing NT gRNA, SCD gRNA 1 and SCD gRNA 2 in the presence of doxorubicin (1 µM). Values are normalized to NT gRNA and expression levels of SCD in all treated groups are shown in representative western blot. (C) Representative western blot (n=3) of 4-hydroxynonenal levels in MV-4-11 cells treated with SSI-4 (1 µM), doxorubicin (1 µM), Ferrostatin-1 (5 µM) and their combinations. (D-E) MV-4-11 cells were treated for 72h with SSI-4 (1 µM) with or without addition of doxorubicin (1 µM) and Ferrostatin-1 (5 µM), FASN inhibitor Fasnall (20 µM) or AMPK activator MK-8722 (10 µM). Cell death was determined by Annexin-V expression. (F) Correlation of SCD expression and sensitivity to cytarabine in samples from BeatAML 2.0 dataset. (G) Percentage of CD45.2+ leukemia blasts in peripheral blood of NBSGW mice before starting treatment. Data are mean ± SD * p < 0.05, ** p < 0.01, *** p < 0.001, **** p < 0.0001.

**Supplemental Data 1. Characteristics of patient samples used in study (Barts Cancer Institute cohort) (excel file)**

**Supplemental Data 2. Characteristics of AML cell lines used in study (excel file)**

**Supplemental Data 3. Lipidomics analysis raw data (excel file)**

**Supplemental Data 4. Fatty acids profiling cell lines raw data (excel file)**

**Supplemental Data 5. Fatty acids profiling primary samples raw data (excel file)**

**Supplemental Data 6. Differentially expressed genes MV-4-11 SSI-4 vs control 24h (excel file)**

**Supplemental Data 7. Differentially expressed genes MV-4-11 SSI-4 + oleate vs SSI-4 24h (excel file)**

**Supplemental Data 8. Enriched datasets used in the study (excel file)**

**Supplemental Data 9. Reagents used in the study**

| **REAGENT** | **MANUFACTURER** | **CATALOGUE NUMBER** |
| --- | --- | --- |
| **Antibodies and dilutions (western blot)** | | |
| SCD Mouse mAb (CD.E10) (1:1000) | ThermoFisher | # MA5-27542, RRID: AB_2723611 |
| FASN Rabbit mAb (C20G5) (1:1000) | Cell Signaling Technology | # 3180, RRID: AB_2100796 |
| ACC Rabbit mAb (C83B10) (1:1000) | Cell Signaling Technology | # 3676, RRID:AB_2219397 |
| AMPKα Rabbit mAb (D5A5) (1:1000) | Cell Signaling Technology | # 5831, RRID:AB_10622186 |
| ph-AMPKα (Thr172) Rabbit mAb (40H9) (1:1000) | Cell Signaling Technology | # 2535, RRID: AB_331250 |
| Cleaved PARP (Asp214) Rabbit mAb (D64E10) (1:1000) | Cell Signaling Technology | # 5625, RRID:AB_10699459 |
| Caspase-3 Rabbit mAb (D3R6Y) (1:1000) | Cell Signaling Technology | # 14220, RRID:AB_2798429 |
| PERK Rabbit mAb (C33E10) (1:1000) | Cell Signaling Technology | # 3192, RRID:AB_2095847 |
| eIF2α Rabbit mAb (D7D3) (1:1000) | Cell Signaling Technology | # 5324, RRID:AB_10692650 |
| Phospho-eIF2α (Ser51) Rabbit mAb (D9G8) (1:1000) | Cell Signaling Technology | # 3398, RRID:AB_2096481 |
| β-Actin Mouse mAb (8H10D10) (1:20 000) | Cell Signaling Technology | # 3700, RRID:AB_2242334 |
| Anti-mouse IgG, HRP-linked Antibody (1:2000) | Cell Signaling Technology | # 7076, RRID:AB_330924 |
| Anti-rabbit IgG, HRP-linked Antibody (1:2000) | Cell Signaling Technology | # 7074, RRID:AB_2099233 |
| SREBP-2 Mouse Ab IgG-1C6 (1:1000) | BD Pharmingen™ | # 557037 RRID:AB_396560 |
| SREBP-1 Mouse mAb (2A4) (1:1000) | AbCam | # ab3259, RRID:AB_303650 |
| FADS2 Rabbit polyclonal Ab (1:1000) | AbCam | # ab232898 |
| 4-hydroxynonenal polyclonal Ab (1:1000) | Alpha Diagnostic Intl. | # HNE11-S |
| **Antibodies and dilutions (flow cytometry)** |  |  |
| Annexin V FITC (1:30) | BioLegend | # 640945 |
| anti-human CD45 Pacific Blue™ (1:50) | BioLegend | # 982306, RRID:AB_2650649 |
| anti-mouse CD45 APC (1:50) | BioLegend | # 157605, RRID:AB_2876537 |
| anti-human CD19 Brilliant Violet 711™ (1:50) | BioLegend | # 302245, RRID:AB_2562062 |
| anti-human CD33 PE (1:50) | BioLegend | # 303404, RRID:AB_314348 |
| anti-mouse CD45.1 Brilliant Violet 711™ (1:50) | BioLegend | # 110739, RRID:AB_2562605 |
| anti-mouse CD45.2 FITC (1:50) | BioLegend | # 109805, RRID:AB_313442 |
| anti-mouse CD117 (c-kit) PE (1:50) | BioLegend | # 135105, RRID:AB_1877216 |
| anti-mouse Ly-6G/Ly-6C (Gr-1) PE/Cyanine7 (1:50, 1:1000) | BioLegend | # 108415, RRID:AB_313380 |
| anti-mouse/human CD11b APC (1:50, 1:1000) | BioLegend | # 101212, RRID:AB_312795 |
| anti-mouse/human CD45R/B220 PerCP (1:100) | BioLegend | # 103233, RRID:AB_893355 |
| anti-mouse CD19 APC/Cyanine7 | BioLegend | # 152411, RRID:AB_2922473 |
| anti-mouse CD8a PE (1:1000) | BioLegend | # 100707, RRID:AB_312746 |
| anti-mouse CD4 PE (1:5000) | BioLegend | # 100407, RRID:AB_312692 |
| Streptavidin Brilliant Violet 421™ (1:200) | BioLegend | # 405225 |
| anti-mouse Lineage Panel biotin (1:5) | BioLegend | # 133307, RRID:AB_11124348 |
| anti-mouse CD117 (c-Kit) Brilliant Violet 711™ (1:100) | BioLegend | # 105835, RRID:AB_2565956 |
| anti-mouse Ly-6A/E (Sca-1) APC/Cyanine7 (1:100) | BioLegend | # 108125, RRID:AB_10639725 |
| anti-mouse CD48 PE (1:250) | BioLegend | # 103405, RRID:AB_313020 |
| anti-mouse CD150 (SLAM) PE/Cyanine7 (1:100) | BioLegend | # 115914, RRID:AB_439797 |
| anti-human CD36 APC (1:20) | BD Pharmingen™ | # 550956, RRID:  AB_398480 |
| anti-human LDLR PE (1:20) | AbCam | # ab275614 |
| Human TruStain FcX™ (Fc Receptor Blocking Solution) | BioLegend | # 422302 RRID:AB_2818986 |
| TruStain FcX™ PLUS (anti-mouse CD16/32) Antibody | BioLegend | # 156604 |
| **Cytokines** | | |
| Recombinant Mouse IL-3 (carrier-free) | BioLegend | # 575504 |
| Recombinant Mouse IL-6 (carrier-free) | BioLegend | # 575704 |
| Recombinant Mouse SCF (carrier-free) | BioLegend | # 579704 |
| Recombinant Mouse G-CSF (carrier-free) | BioLegend | # 574604 |
| Recombinant Human G-CSF (carrier-free) | BioLegend | # 578604 |
| Recombinant Human IL-3 (carrier-free) | BioLegend | # 578004 |
| Recombinant Human TPO (carrier-free) | BioLegend | # 763704 |
| **Bacterial and virus strains** | | |
| E.coli DH5α | Kind gift of B. Huntly | NCBI:txid668369 |
| psPAX2 | Addgene | #12260, RRID:Addgene_12260 |
| pMD2.G | Addgene | #12259, RRID:Addgene_12259 |
| MSCV-Hoxa9-neo | Kind gift of T. Sommerville | NA |
| MSCV-Meis1a-puro | Kind gift of T. Sommerville | NA |
| **Chemicals** | | |
| Propidium iodide solution | Sigma-Aldrich | # P4864 |
| TO-PRO-3 | Life Technologies | # T3605 |
| 7-AAD | BioLegend | # 420404 |
| Annexin Binding Buffer | bioWORLD | # 21720002 |
| Ammonium Chloride Solution | Stemcell Technologies | # 07850 |
| SSI-4 | Modulation Therapeutics Inc. | NA |
| A939572 | Sigma-Aldrich | # SML2356 |
| Sodium palmitate | Sigma-Aldrich | # P9767 |
| Sodium oleate | Sigma-Aldrich | # O7501 |
| Fasnall (benzenesulfonate) | Cayman Chemical | # 19957 |
| PF-05175157 | MedChemExpress | # HY-12942 |
| MK-8722 | MedChemExpress | # HY-111363 |
| Ferrostatin-1 | MedChemExpress | # HY-100579 |
| Liproxstatin-1 | MedChemExpress | # HY-12726 |
| Q-VD-OPh | MedChemExpress | # HY-12305 |
| GSK2656157 | Sigma-Aldrich | # 504651 |
| 4μ8C | Sigma-Aldrich | # SML0949 |
| Doxorubicin hydrochloride | Sigma-Aldrich | # D1515 |
| 1-β-D-Arabinofuranosylcytosine (cytarabine) | Sigma-Aldrich | # 251010 |
| Puromycin dihydrochloride | Sigma-Aldrich | # P8833 |
| Doxycycline hyclate | Sigma-Aldrich | # D5207 |
| Sucrose | Sigma-Aldrich | # 573113 |
| D-GLUCOSE (U-13C6, 99%) | Cambridge Isotope Laboratories Inc. | # CLM-1396-PK |
| Insulin solution human (10 mg/mL) | Sigma-Aldrich | # I9278 |
| NuPAGE™ 4 to 12%, Bis-Tris, 1.0–1.5 mm, Mini Protein Gels | Invitrogen™ | # NP0321BOX |
| NuPAGE™ 3 to 8%, Tris-Acetate, 1.0–1.5 mm, Mini Protein Gels | Invitrogen™ | # EA0375BOX |
| NuPAGE™ LDS Sample Buffer (4X) | Invitrogen™ | # NP0007 |
| NuPAGE™ MES SDS Running Buffer (20X) | Invitrogen™ | # NP0002 |
| NuPAGE™ Tris-Acetate SDS Running Buffer (20X) | Invitrogen™ | # LA0041 |
| NuPAGE™ Transfer Buffer (20X) | Invitrogen™ | # NP00061 |
| Immobilon®-P PVDF Membrane | Millipore | # IPVH00005 |
| Novex™ Sharp Pre-stained Protein Standard | Invitrogen™ | # LC5800 |
| PageRuler™ Plus Prestained Protein Ladder, 10 to 250 kDa | ThermoFisher™ | # 26619 |
| Protease Inhibitor Cocktail Set I | Sigma-Aldrich | # 539131 |
| Phosphatase Inhibitor Cocktail Set III | Millipore | # 524627 |
| Clarity Western ECL Substrate | BioRad | # 1705061 |
| SuperSignal™ West Pico PLUS Chemiluminescent Substrate | ThermoFisher™ | # 34579 |
| EasySep™ Human TCR Alpha/Beta Depletion Kit | StemCell Technologies | # 17847 |
| TransIT®-Lenti Transfection Reagent | Mirus Bio | # MIR 6603 |
| Polybrene Infection / Transfection Reagent | Sigma-Aldrich | # TR-1003 |
| **Assays** | | |
| Zombie NIR™ Fixable Viability Kit | BioLegend | # 423106 |
| BODIPY™ 581/591 C11 (Lipid Peroxidation Sensor) | Invitrogen™ | # D3861 |
| C1- BODIPY™ 500/510 C12 (Lipid Uptake Sensor) | Invitrogen™ | # D3823 |
| Direct-zol RNA Microprep | Zymo Research | # R2061 |
| High-Capacity cDNA Reverse Transcription Kit | Applied Biosystems™ | # 4368814 |
| PowerUp™ SYBR™ Green Master Mix | Applied Biosystems™ | # A25741 |
| EndoFree Plasmid Maxi Kit (10) | Qiagen | # 12362 |
| **Oligonucleotides** | | |
| SCD gRNA 1: 5’-CACCGATATATGACCCCACCTACA-3’ | Sigma-Aldrich | NA |
| SCD gRNA 2: 5’-CACCGCATATTCAACCTTGGGGCT-3’ | Sigma-Aldrich | NA |
| NT gRNA: 5’-CACCGATTTTCGTACCCTGGGACGC-3’ | Sigma-Aldrich | NA |
| pKLV2 primer: 5’-AGATAATTAGAATTAATTTGACTG-3’ | Sigma-Aldrich | NA |
| **Recombinant DNA** | | |
| lentiCas9-Blast | Addgene | #52962, RRID:Addgene_52962 |
| pKLV2-U6gRNA5(BbsI)-PGKpuro2ABFP-W | Addgene | #67974, RRID:Addgene_67974 |
| **Media** | | |
| MyeloCult™ H5100 | StemCell Technologies | # 05150 |
| MethoCult™ GF M3434 | StemCell Technologies | # 03434 |
| MethoCult™ M3231 | StemCell Technologies | # 03231 |
| RPMI | Gibco™ | # 11875093 |
| DMEM | Gibco™ | # 11965092 |
| IMDM | Gibco™ | # 12440053 |
| MEM-α | Gibco™ | # 12571063 |
| OptiMEM | Gibco™ | # 31985070 |

**Supplemental Data 10. Primers for human cDNA (qPCR) used in the study**

|  | **Forward** | **Reverse** |
| --- | --- | --- |
| **ATF4** | GCTAAGGCGGGCTCCTCCGA | ACCCAACAGGGCATCCAAGTCG |
| **ATF6** | ATGAAGTTGTGTCAGAGAACC | CTCTTTAGCAGAAAATCCTAG |
| **CHOP** | GGAGCATCAGTCCCCCACTT | TGTGGGATTGAGGGTCACATC |
| **IRE 1** | AGTCAGTTCTGCGTCCGCT | TGGTACTTCCAAAAATCCCGAGG |
| **PERK** | ATGCTTTCACGGTCTTGGTC | TCATCCAGCCTTAGCAAACC |
| **XBP1 spliced** | TTGCTGAAGAGGAGGCGGAA | CTGCACCTGCTGCGGACTCAG |
| **XBP1 total** | TTCCGGAGCTGGGTATCTCA | GAAAGGGAACCCCCGTATCC |
| **MIR22HG** | CCTCGTGCAGCAACCCC | GTGAGGGCGTGAGAGGAAC |
| **actB** | GCCGCCAGCTCACCAT | TCGTCGCCCACATAGGAATC |
